# Deconstructing the RAGE signaling maze: the molecular key to opening a new dimension of ovarian anti-aging

**DOI:** 10.1038/s12276-026-01678-3

**Published:** 2026-04-20

**Authors:** Xia Bai, Guohui Zhang, Xiao Xiao, Qin Zeng, Yuhong Zhao, Pinghan Wang, Fangyi Long, Weixin Liu

**Affiliations:** 1 Laboratory Medicine Center, Sichuan Provincial Women’s and Children’s Hospital/The Affiliated Women’s and Children’s Hospital of Chengdu Medical College, Chengdu, China; 2 Center for Reproductive Medicine, Sichuan Provincial Women’s and Children’s Hospital/The Affiliated Women’s and Children’s Hospital of Chengdu Medical College, Chengdu, China

**Keywords:** Mechanisms of disease, Infertility

## Abstract

The ovaries are vital components of the female reproductive system. Ovarian aging, driven by oxidative stress, chronic inflammation and hormonal dysregulation, severely compromises female fertility. The receptor for advanced glycation end products (RAGE) serves as a critical regulator of ovarian physiology and pathology. linking metabolic dysfunction to reproductive decline. This Review synthesizes evidence that RAGE hyperactivation, during the process of ovarian aging, disrupts folliculogenesis, granulosa cell function and steroidogenesis via MAPK-ERK, PI3K-AKT-mTOR and NF-κB pathways, exacerbating conditions such as premature ovarian failure, polycystic ovary syndrome and ovarian cancer. Furthermore, we summarizes existing therapeutic strategies targeting RAGE and underscores their potential in mitigating ovarian aging and treating ovarian pathologies, providing novel perspectives for preserving female reproductive capacity. We highlight therapeutic strategies targeting RAGE, including small-molecule inhibitors (Azeliragon and FPS-ZM1), soluble RAGE decoys and natural compounds, which show promise in restoring ovarian reserve and hormonal balance in preclinical models. These interventions mitigate advanced glycation end products (AGE)–RAGE-induced damage, offering novel avenues to preserve fertility. Beyond reproductive health, RAGE’s role in aging and metabolic disorders underscores its potential as a cross-disciplinary biomarker and therapeutic target. By bridging molecular mechanisms with clinical applications, this work provides a framework for developing precision therapies to combat ovarian aging, with implications for endocrinology, oncology and geroscience.

## RAGE hyperactivation disrupts ovarian function and fertility

The Review Article discusses the challenges of declining fertility rates and aging populations worldwide. It highlights how age-related changes in the ovaries contribute to these issues. We focus on a protein called receptor for advanced glycation end products (RAGE), which plays a role in ovarian aging and related diseases. The study reviews how RAGE affects ovarian function, including hormone production and follicle development. It explains that RAGE overexpression can lead to inflammation, oxidative stress and cell death, worsening conditions such as premature ovarian failure and polycystic ovary syndrome. Researchers also explore potential treatments targeting RAGE, such as small molecule inhibitors and antibodies. The findings suggest that targeting RAGE could help manage ovarian aging and related disorders. Future implications include the potential for new treatments to improve fertility and delay ovarian aging by focusing on RAGE pathways.

This summary was initially drafted using artificial intelligence, then revised and fact-checked by the author.

## Facts

- RAGE-induced oxidative stress reduces follicular quality in aging ovary.
- RAGE induces granulosa cell dysfunction: autophagic disruption and hormonal dysregulation.
- RAGE causes fibrosis of the ovarian stroma, integrating changes in the ovarian microenvironment.
- RAGE inhibitors hold promise for treating age-related ovarian decline.

## Open questions

- What is the core mechanism of RAGE overexpression leading to ovarian aging?
- Can tissue-specific RAGE inhibition strategies overcome the limitations of systemic RAGE antagonists to effectively delay ovarian aging while preserving physiological RAGE functions in other organs?
- Why does RAGE overexpression manifest as divergent pathologies and do ligand-specific interactions or genetic variants dictate these distinct clinical phenotypes?

## Introduction

The global demographic transition has been marked by a precipitous decline in fertility rates, synergistically driving accelerated population aging that presents unprecedented challenges to global socioeconomic sustainability^1^. This fertility recession arises from a complex interplay of sociobehavioral and biological determinants, where declining reproductive autonomy intersects with an age-related decline in the ovarian reserve. From a biological perspective, maternal aging is intrinsically linked to progressive ovarian functional decay characterized by diminished follicular quantity/quality and altered endocrine dynamics, establishing a finite window of reproductive competence.

**Fig. 1:**
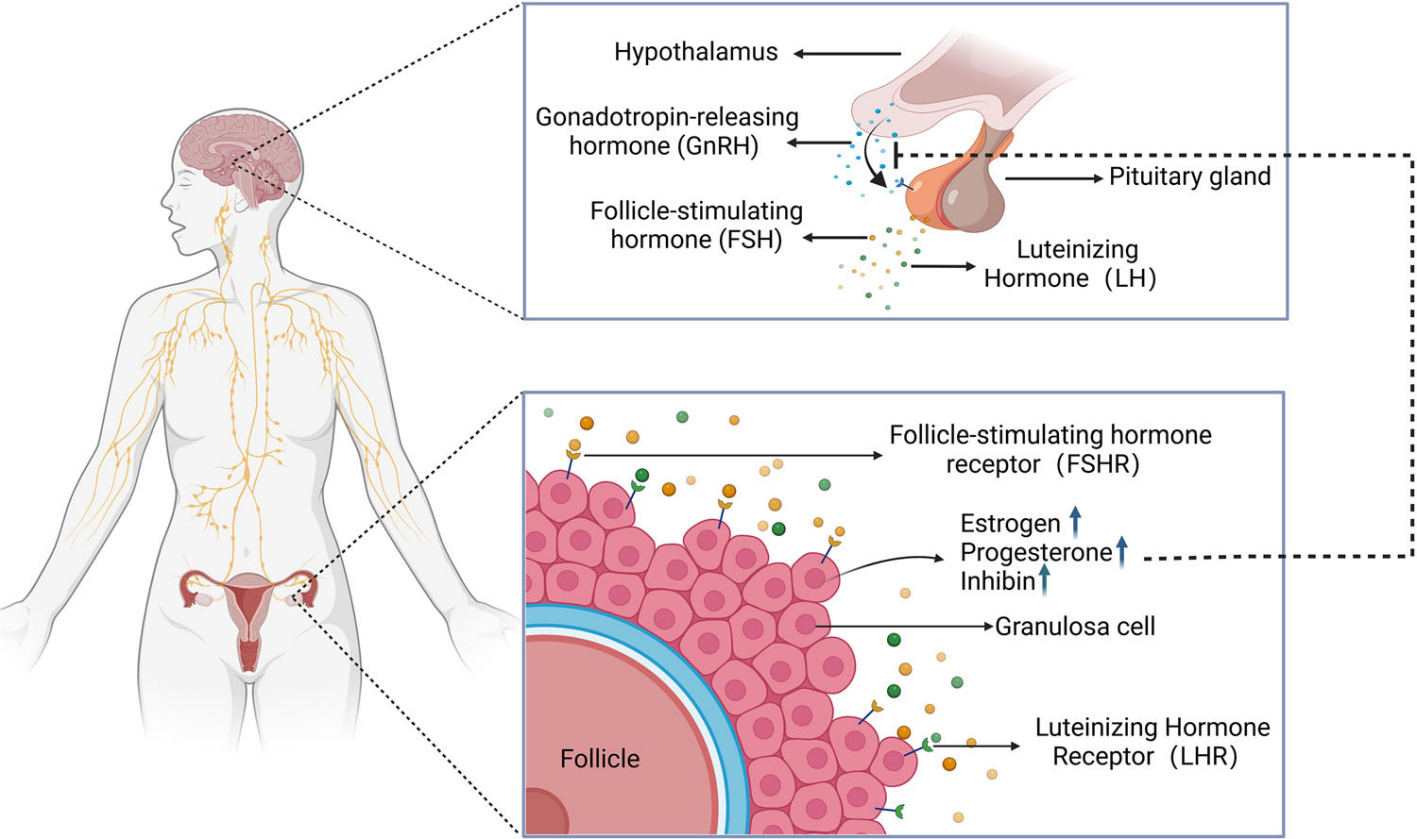
Schematic diagram of HPO axis. The HPO axis uses pulsatile GnRH to control pituitary LH/FSH release, which bind ovarian receptors (LHR/FSHR) to trigger folliculogenesis and steroid production. Sex hormones feedback to suppress GnRH/gonadotropins, while estrogen briefly boosts GnRH mid-cycle. Inhibin also selectively lowers FSH, synchronizing hormone cycles. Image created with BioRender; https://www.biorender.com/.

Ovaries regulate female reproduction through gametogenic/steroidogenic functions^2^. Cyclical oocyte maturation requires coordinated nuclear/cytoplasmic development, determining reproductive success. Postovulatory transport facilitates spermoocyte fusion and embryogenesis via blastocyst development/implantation^3,4^. The ovarian endocrine axis secretes dynamic hormones (estradiol, progesterone and inhibins) under hypothalamic–pituitary–ovarian (HPO) axis regulation, involving multilevel feedback^5^. Pulsatile gonadotropin-releasing hormone (GnRH) stimulates pituitary follicle-stimulating hormone (FSH)/luteinizing hormone (LH) production^6^, with FSH driving follicular maturation and LH surge inducing ovulation/corpus luteum formation^7^. Steroid hormones modulate GnRH/gonadotropin secretion via feedback loops^8^. Molecular studies reveal HPO regulation through kisspeptinergic pathways, AMH dynamics and growth factor interactions^9^ (Fig. 1).

Notably, the pathophysiology of age-related ovarian senescence manifests through multifactorial desynchronization of gonadotropic feedback mechanisms, encompassing both quantitative depletion of primordial follicle reserves and qualitative alterations in gonadotropin receptor responsiveness—representing fundamental pathophysiological mechanisms underlying the progressive decline in fecundity observed in advanced reproductive aging^10^. The physiological process of ovarian aging is characterized by progressive depletion of the primordial follicle reserve, concomitant with reactive oxygen species (ROS)-mediated oxidative stress that induces mitochondrial DNA (mtDNA) damage^11,12^. This dual mechanism ultimately culminates in oocyte apoptosis and functional impairment of granulosa cells. Simultaneously, the aging ovarian microenvironment sustains a persistent low-grade inflammatory milieu, evidenced by elevated proinflammatory cytokine production that drives stromal fibrosis and microvascular degeneration^13–15^. At the molecular level, granulosa cells exhibit downregulation of steroidogenic enzymes such as aromatase and 3β-hydroxysteroid dehydrogenase, precipitating diminished estrogen and progesterone synthesis that underlies menopausal symptomatology^16^. Notably, ovarian pathophysiological conditions have emerged as critical determinants of global fertility decline. Polycystic ovary syndrome (PCOS), with a worldwide prevalence of 6-20%, induces anovulatory infertility through endocrine disruption-mediated impairment of folliculogenesis and oocyte developmental competence^17,18^. Premature ovarian failure (POF), affecting approximately 1% of reproductive-aged women, depletes the primordial follicle pool decades before physiological menopause, substantially reducing the natural conception rates of affected women under 40 years^19^. Importantly, iatrogenic interventions for ovarian malignancies, including surgical oophorectomy and chemoradiotherapy, cause irreversible ovarian dysfunction in patients through direct follicular destruction and stromal damage^20,21^. These disorders share common pathogenic features of oocyte quality deterioration, endocrine axis disruption and ovarian microenvironment alterations. When superimposed on environmental endocrine disruptors and shifting reproductive demographics toward advanced maternal age, these factors synergistically contribute to the current infertility pandemic.

In recent years, receptor for advanced glycation end products (RAGE) has emerged as a critical signaling molecule regulating key ovarian functions, including hormone secretion, follicular development and ovulation. RAGE, a pivotal pattern recognition receptor in the innate immune system, exhibits multiligand binding properties that enable recognition of diverse damage-associated molecular patterns (DAMPs) including advanced glycation end products (AGEs), high mobility group box 1 protein (HMGB1) and S100 calcium binding protein family members^22^. Upon activation, this receptor initiates cascade signaling through multiple pathways such as nuclear factor-κB (NF-κB), mitogen-activated protein kinase (MAPK) and transforming growth factor β (TGF-β)-Smad, mediating inflammatory responses, oxidative stress injury and programmed cell death^23,24^. Under physiological conditions, the RAGE signaling network contributes to tissue homeostasis and host defense mechanisms^25^. However, in chronic pathological states such as diabetes mellitus and atherosclerosis, persistent ligand stimulation induces pathological hyperactivation of the receptor, establishing a deleterious positive feedback loop that drives proinflammatory cytokine storms, excessive ROS accumulation, and cellular dysfunction^26^. Notably, in ovarian pathologies including POF, PCOS and ovarian carcinoma, RAGE overexpression has been observed to exert multipathway detrimental effects: compromising oocyte integrity through cell cycle dysregulation and impaired maturation, inducing granulosa cell dysfunction, disrupting ovarian steroidogenesis by inhibiting steroidogenic enzyme conversion and potentially accelerating ovarian tissue fibrosis through extracellular matrix (ECM) remodeling^27–29^. Therefore, this Review aims to systematically clarify the pathological processes and molecular mechanisms by which RAGE overactivation accelerates ovarian aging and the occurrence of ovarian-related diseases. By integrating the latest research evidence, it systematically analyzes the potential molecular mechanisms of RAEG and the multidimensional signaling pathway networks, and develops new biomarkers for ovarian aging.

## RAGE

Moaddel et al. conducted comprehensive enrichment analysis of 232 age-related proteins across biological models, revealing the AGE–RAGE signaling pathway as one of the most statistically pronounced metabolic pathways associated with biological aging^30^. This finding aligns with established mechanisms of ovarian aging characterized by declining antioxidant capacity and cumulative oxidative damage (as referenced in oxidative stress studies of ovarian aging). Notably, accumulating evidence demonstrates that dysregulation of RAGE expression exhibits marked correlations with various ovarian pathologies, particularly those involving folliculogenesis abnormalities and steroidogenic dysfunction, consistent with the observed deterioration of ovarian reserve during reproductive senescence.

In 1992, Schmidt et al. at Columbia University identified RAGE, a 35–55 kDa transmembrane protein belonging to the immunoglobulin superfamily, as a receptor for AGEs^31,32^. RAGE functions as a multiligand pattern recognition receptor, binding diverse molecules including AGEs, S100 proteins, HMGB1 and amyloid β-peptide^33–35^. Structurally, RAGE contains an extracellular domain with three immunoglobulin-like regions (V, C1 and C2) for ligand binding, a transmembrane α-helix and a cytoplasmic signaling tail^31^. The V domain mediates primary ligand interactions, while C1/C2 domains stabilize the binding pocket. Glycosylation sites and disulfide bonds in the extracellular domain regulate receptor stability and function^36–39^. RAGE activation drives inflammatory, oxidative and metabolic pathways in disease pathogenesis.

RAGE has multiple splice variants, with the full-length transmembrane form being the most studied. Two soluble isoforms exist: endogenous secretory RAGE (esRAGE, also known as AQ7RAGE) and shedded soluble RAGE (sRAGE) generated by ectodomain cleavage. Both isoforms retain the extracellular V, C1 and C2 domains but lacks transmembrane and cytoplasmic regions, though they originate from distinct molecular mechanisms^40,41^. sRAGE arises via alternative splicing or proteolytic cleavage of membrane-bound RAGE by matrix metalloproteinases (MMPs)/ADAMs^42,43^. Functionally, sRAGE acts as a decoy receptor, sequestering ligands (AGEs) to inhibit inflammatory signaling and oxidative damage^44,45^. The soluble pool includes endogenous sRAGE (splicing-derived, unique C-terminus) and cleaved RAGE (cRAGE) (proteolytically shed from full-length RAGE)^46,47^. Both isoforms exhibit similar ligand-binding functions but differ in biogenesis.

**Fig. 2:**
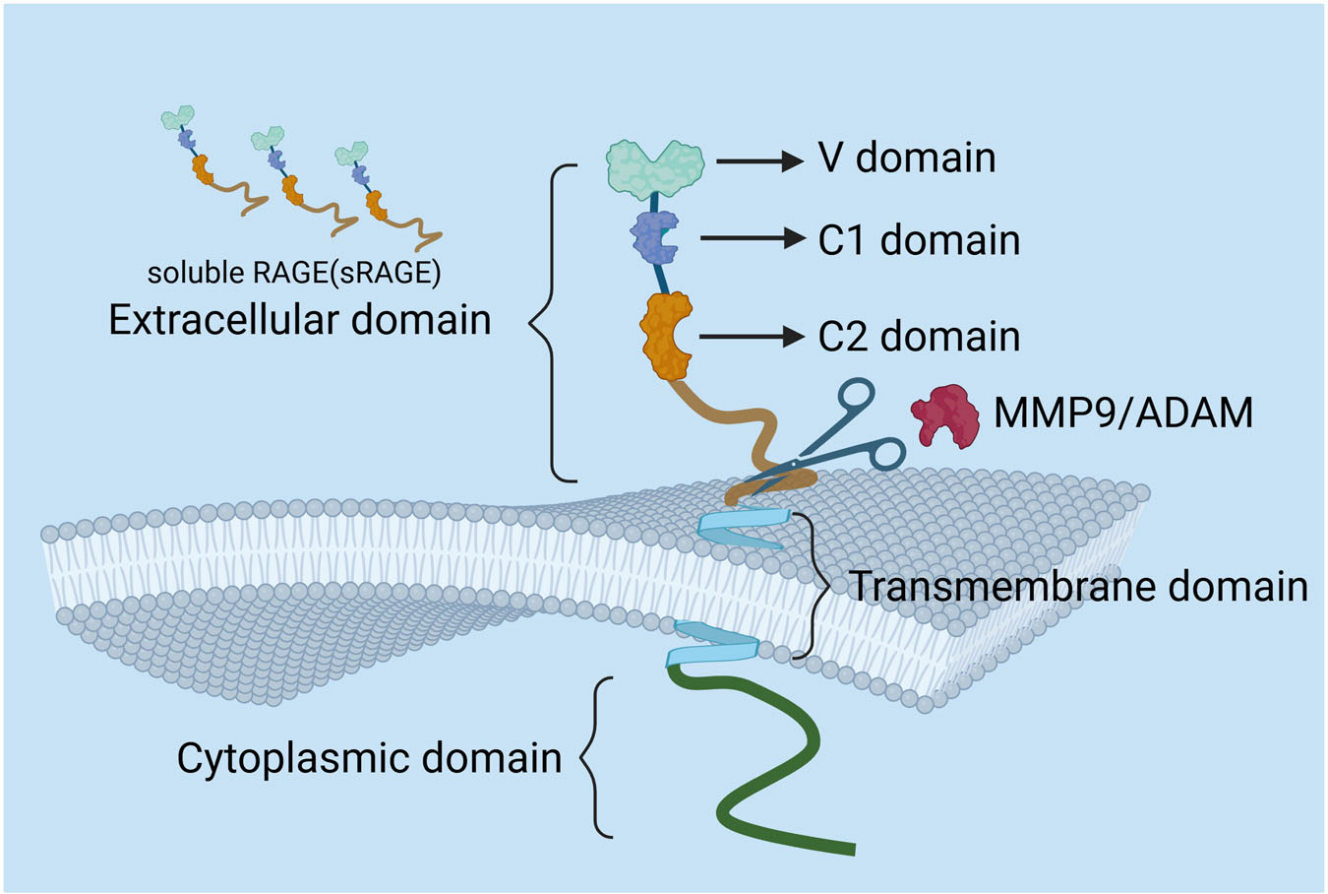
Schematic diagram of the structure of RAGE and sRAGE. RAGE has a three-part structure: an N-terminal V domain for ligand binding, two C domains (C1 and C2), a transmembrane helix and a signaling tail. Proteases cleave it to release sRAGE, keeping V–C1–C2 but losing membrane parts. Image created with BioRender; https://www.biorender.com/.

RAGE displays spatiotemporal expression patterns with key roles in embryogenesis and tissue homeostasis, showing dynamic regulation in pathology. It is predominantly expressed in endothelial cells, smooth muscle cells, immune cells, neurons and cancer cells^48–51^. While constitutively expressed in adult lung tissue, pathological conditions induce cytoplasmic RAGE in prostate, testicular and urothelial carcinomas^52,53^. Developmental studies show high RAGE levels in pulmonary and neural tissues during organogenesis, with postnatal downregulation^54^ (Fig. 2).

## RAGE regulation of ovarian physiological function

In ovarian tissue, RAGE is expressed in immune cells, theca interna cells, granulosa cells, stroma and vascular smooth muscle, with comparable levels in granulosa and theca cells^55^. Ovarian-specific isoforms include follicular fluid sRAGE^56^ and a novel 12-amino acids peptide (RAGE_344-355_) in PCOS oocytes^57^, demonstrating context-dependent molecular diversity.

**Fig. 3:**
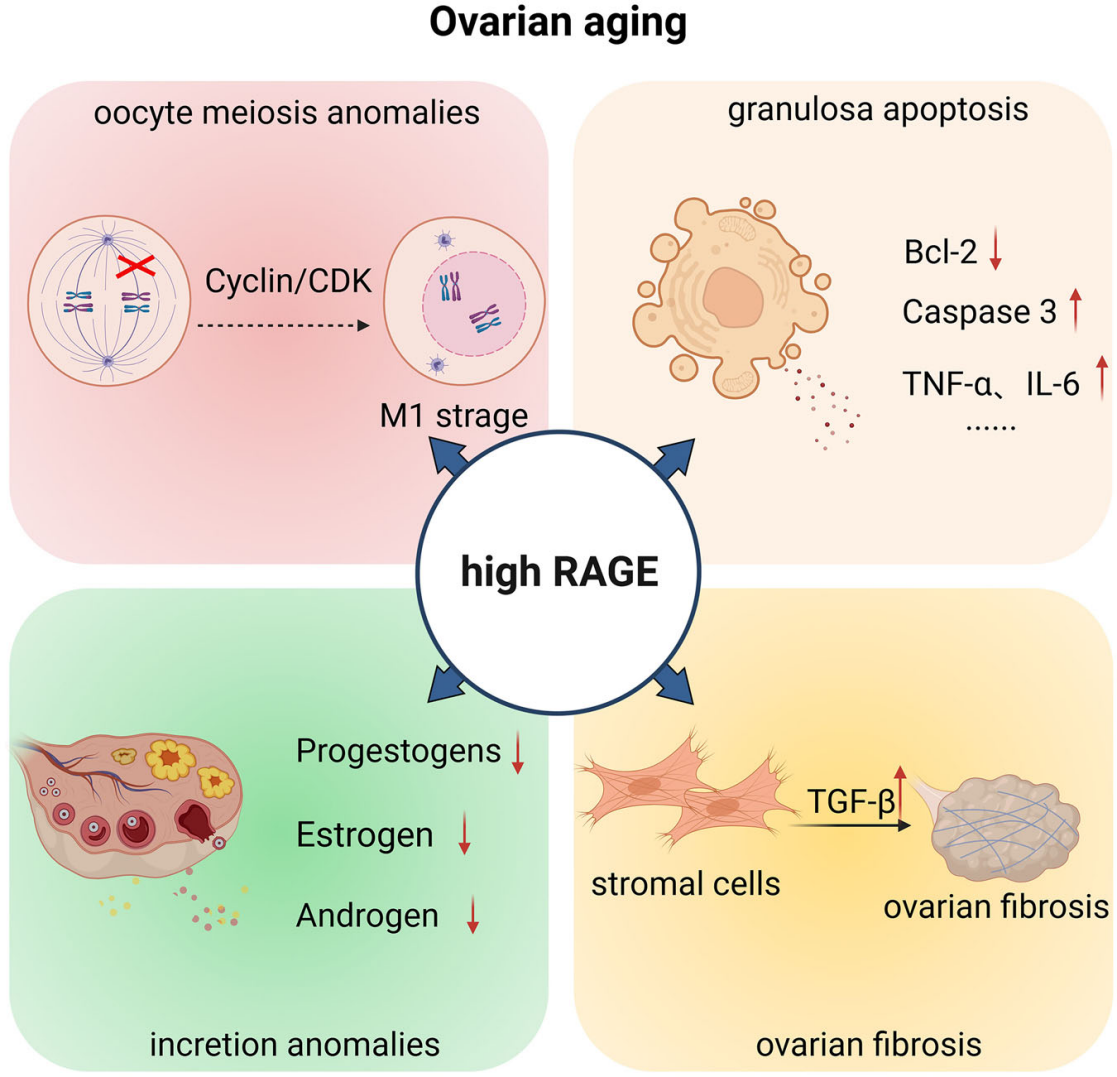
The upregulation of RAGE expression in aging ovaries appears to exert multifaceted impacts on ovarian physiology through distinct molecular pathways. High RAGE levels disrupt oocyte meiosis by impairing spindle assembly, causing metaphase I arrest. RAGE overexpression in granulosa cells triggers apoptosis via caspase pathways, promotes proinflammatory cytokine secretion, disrupts steroid hormone synthesis by reducing key enzymes and induces fibrosis via TGF-β-mediated collagen deposition. Image created with BioRender https://www.biorender.com/.

Under normal physiological conditions, RAGE demonstrates basal expression levels in ovarian tissue. Age-related or pathological stimuli processes leading to RAGE overexpression have been mechanistically intertwined with ovarian dysfunction through multiple interconnected pathways: (1) disruption of meiotic progression and impaired regulation of terminal oocyte maturation; (2) dysregulation of granulosa cell proliferation–apoptosis dynamics coupled with enhanced proinflammatory cytokine secretion, consequently compromising ovarian microenvironmental homeostasis; (3) attenuation of steroidogenic hormone biosynthesis and perturbation of the physiological equilibrium between corpus luteum formation and programmed regression (Fig. 3).

### RAGE disrupts oocyte meiotic progression and impaires follicular development in aging ovarian

The biological essence of ovarian aging manifests as an irreversible decline in both the quantity and quality of ovarian follicles, characterized by multilevel pathophysiological alterations encompassing impaired folliculogenesis, meiotic recombination abnormalities and microenvironmental homeostasis disruption. Current research^58^ reveals that oocyte maturation is critically modulated by follicular fluid components, including steroid hormones, bioactive metabolites, and proteomic signatures. Notably, RAGE overexpression has been detected in the follicular fluid of aging females. The AGE–RAGE ligand–receptor interaction potentially induces vascular endothelial growth factor (VEGF) overexpression, suggesting this biochemical milieu might serve as a biomarker for reproductive dysfunction during ovarian senescence^59^.

#### RAGE modulates oocyte cell cycle progression and orchestrates DNA repair pathways

The precise regulation of cyclins and cyclin-dependent kinases (CDKs) is crucial for ensuring the orderly progression of oocytes through successive developmental stages^60^. During folliculogenesis, oocyte survival and follicular atresia are markedly influenced by the phosphatidylinositol 3-kinase (PI3K)-protein kinase B (AKT) signaling pathway^61^. Research has demonstrated that the receptor for RAGE modulates the PI3K-AKT-mammalian target of rapamycin (mTOR) pathway in periodontal ligament cells. This regulation impacts cellular viability and the expression of key autophagy markers, including autophagy-related protein 5 (ATG5), Beclin-1 and the microtubule-associated protein light chain 3 (LC3-II/LC3-I) ratio. Furthermore, these changes affect the expression of cell cycle regulatory proteins (cyclinD1-CDK4 and cyclinA-CDK2 complexes), ultimately leading to cell cycle dysregulation and enhanced inflammatory responses^62,63^.

The overexpression of RAGE in aging ovaries appears to compromise oocyte DNA repair capacity through modulation of the PI3K-Akt signaling pathway^64^. This pathway serves as a crucial regulator in the molecular mechanisms underlying DNA damage repair and oocyte quality maintenance. Notably, PI3K-related protein kinases (PIKKs) are recognized as key regulators of cellular DNA damage response systems. Mechanistically, Akt activation engages cell cycle checkpoint kinase 1 (Chk1), a critical component of the DNA damage response system. This kinase mediates cell cycle arrest during S and G2 phases, allowing for error correction prior to mitotic entry^65^. However, aberrant PI3K-Akt signaling has been shown to impair DNA double-strand break repair through multiple mechanisms: inactivation of the G2/M checkpoint, phosphorylation-mediated suppression of Chk1 activity and cytoplasmic sequestration of breast cancer susceptibility gene 1 (BRCA1) protein^66^.

Notably, RAGE overexpression induces hyperactivation of Akt in mature oocytes, resulting in dual regulatory effects: upregulation of cell proliferation-related genes concurrent with downregulation of proapoptotic factors including B cell lymphoma-2 (BCL-2), BCL-2-associated X protein (BAX) and caspase-3^67^. Emerging evidence suggests that Akt orchestrates apoptotic regulation in oocytes through three distinct mechanisms: modulation of proapoptotic protein expression, control of subcellular localization and stabilization of endogenous apoptosis inhibitors^68^.

#### RAGE-induced oxidative stress directly impairs oocyte mitochondrial function and DNA integrity

Ovarian aging is primarily driven by the accelerated depletion of follicular reserve, a process that intensifies notably after the age of 30. The accumulation of AGEs and activation of their receptor RAGE, may critically mediate this phenomenon. RAGE signaling can activate the transcription factor NF-κB, which upregulates proinflammatory gene expression and stimulates ROS production in ovarian tissues^69^. Notably, age-associated upregulation of p38 MAPK activity may further amplify oxidative damage via MAPKAPK-2-mediated transcription of proinflammatory cytokines^70^. Oocytes exhibit inherent vulnerability to oxidative stress due to their high lipid content, particularly polyunsaturated fatty acids, rendering them prone to ROS-induced damage^71^. Such dysregulation ultimately contributes to age-related declines in fertility by compromising cumulus-oocyte communication and meiotic arrest resolution.

Overexpression of RAGE may further exacerbate ROS generation by activating intracellular enzymes such as NADPH oxidase. Excessive ROS accumulation exerts multifaceted detrimental effects on oocytes. First, oxidative damage to cellular macromolecules—including DNA, proteins and lipids—disrupts normal cellular function. Specifically, DNA damage in oocytes may lead to chromosomal abnormalities during meiosis, impairing embryo development. Second, ROS-induced mitochondrial dysfunction critically impacts energy metabolism. As mitochondria are central to ATP production in oocytes, compromised mitochondrial activity reduces energy supply, thereby hindering oocyte growth, maturation and increasing susceptibility to apoptotic degeneration^72^.

#### RAGE regulates the gene expression pattern of oocytes

Given the central role of the MAPK-extracellular signal-regulated kinases (ERK) signaling pathway in regulating oocyte maturation and function, this cascade constitutes the primary signaling mechanism through which gonadotrophic hormones (LH and FSH) exert their regulatory effects on ovulation^73^. During aging, the accumulation of AGEs in the ovarian microenvironment exacerbates oxidative stress and disrupts redox homeostasis. AGE–ligand interactions with their receptor RAGE trigger a proinflammatory signaling cascade, promoting hyperphosphorylation of p38 MAPK and elevated phosphorylation of ERK1/2^74^. This aberrant activation of MAPK signaling disrupts spatiotemporal regulation of gene expression in oocytes, as phosphorylated ERK translocates to the nucleus to modulate transcription of genes critical for oocyte metabolism (mitochondrial biogenesis), cytoskeletal dynamics (microtubule assembly) and meiotic progression^75^.

In summary, in the elderly ovaries, the overexpression of RAGE may modulate the PI3K-AKT-mTOR pathway, dysregulating cyclin–CDK complexes and cell cycle checkpoints (Chk1 and BRCA1), thereby compromising DNA repair capacity and increasing genomic instability. Concurrently, RAGE hyperactivates oxidative stress via NF-κB and p38-MAPK signaling, inducing ROS-mediated mitochondrial dysfunction, lipid peroxidation and DNA damage. This redox imbalance further disrupts MAPK-ERK signaling, altering gene expression critical for oocyte metabolism and meiosis. Collectively, RAGE-mediated dysregulation of key signaling pathways and oxidative stress collectively impair oocyte quality, folliculogenesis and fertility in aging ovaries.

### RAGE modulates oxidative stress and autophagy in aging ovarian granulosa cells

Granulosa cells are a type of somatic cell that surrounds the oocyte in the ovarian follicle. They play crucial roles in folliculogenesis, oocyte development and ovulation^76^. Granulosa cells produce various hormones, including estrogen and progesterone, which regulate the female reproductive cycle^77^.

#### RAGE regulates granulosa cells senescence and apoptosis through oxidative stress

RAGE is highly expressed in ovarian granulosa cells in aging women and induces oxidative stress^78^. Following RAGE activation, the p38 MAPK-JNK signaling axis undergoes phosphorylation and subsequent nuclear translocation. Within the nucleus, these kinases phosphorylate and activate transcription factors including ATF-2, c-Jun, CREB and NF-κB^79^. These activated transcription factors assemble into AP-1 complexes that drive transcriptional activation of proinflammatory mediators such as TNF-α, IL-1β, IL-6 and COX-2^79,80^. The synthesized inflammatory factors are subsequently secreted via the canonical secretory pathway, initiating inflammatory cascades. Notably, p38 MAPK extends the temporal expression of inflammatory mediators through mRNA stabilization, while JNK enhances transcriptional activity through post-translational modification of transcription factors^81^. This dual regulatory mechanism creates a synergistic amplification of inflammatory responses, which has been implicated in the pathogenesis of chronic inflammatory conditions including diabetic complications, atherosclerosis and neurodegenerative disorders. In mitochondria, JNK modulates apoptotic signaling through phosphorylation of Bcl-2 family proteins (Bim and Bad), thereby antagonizing the anti-apoptotic functions of Bcl-2 and Bcl-xL^82^. This post-translational modification increases mitochondrial membrane permeability, facilitating cytochrome c release. The liberated cytochrome c forms an apoptosome complex with Apaf-1 and caspase-9, leading to sequential activation of caspase-9 and executioner caspase-3^83^. Concurrently, JNK upregulates Fas receptor expression, thereby sensitizing cells to extrinsic apoptotic signaling^84^. After Fas binds to FasL, Fas-associated death domain protein (FADD) and caspase-8 are recruited to form a death-induced signaling complex (DISC). Caspase-8 is activated, which in turn activates caspase-3, triggering cell apoptosis. Caspase-3 activates CAD (caspase activated DNA enzyme), causing DNA breakage. Caspase-3 cleaves cytoskeletal proteins such as actin and keratin, causing cell shrinkage and membrane blisters. The ultimate apoptotic somas form and are engulfed by adjacent cells or macrophages^85^.

The activation of NF-κB contributes to the upregulation of pyroptosis in granulosa cells, and this phenomenon may be positively correlated with RAGE expression levels^86^. RAGE may upregulate COX-2 and prostaglandin E2 (PGE2) by activating NF-κB^87,88^, thereby interfering with granulomic-oocyte communication, resulting in ovulation dysfunction. RAGE inhibitors (such as sRAGE or anti-RAGE antibodies) or NF-κB inhibitors (such as BAY11-7082) markedly reduce inflammatory factor (IL-6 and TNF-α) levels and reduce apoptosis (by inhibiting Bax and restoring Bcl-2 expression)^89^. RAGE inhibitors can overcome the obstacle of follicle development obstacle^90^.

However, a study indicated that in vitro cultured granulosa-lutein cells exposed to AGE-BSA failed to activate NF-κB signaling or apoptosis, suggesting that AGE-BSA is not a physiological ligand that regulats granulosa cell survival^91^. To this end, they offered several explanations. The cellular effects of AGEs may be related to the internalization of RAGE-binding ligands and vary according to the endocytic capacity of the cells; alternatively, AGEs can bind to multiple cell surface proteins, and their cellular effects may depend on the expression profile of multiple cell receptors^92^. The role of RAGE in regulating the physiological functions of granulosa cells through NF-κB signaling requires further validation. In-depth investigations into the interaction mechanisms between RAGE and NF-κB will increase our understanding of how RAGE modulates ovarian granulosa cells under various physiological and pathological conditions, such as ovulation dysfunction and follicular developmental disorders.

#### Overexpressed RAGE disrupts granulosa cells autophagy, leading to apoptosis

Age-associated overexpression of RAGE may excessively inhibit autophagy through abnormal activation of the PI3K-AKT-mTOR signaling pathway^93^, impairing the timely clearance of damaged organelles and proteins in granulosa cells. The PI3K-AKT pathway plays a crucial role in regulating granulosa cell growth and apoptosis during follicular development. Autophagy and apoptosis appear to coexist in oocytes and granulosa cells, possibly regulating oocyte survival and follicular atresia during follicle formation^94^, and the PI3K-AKT signaling pathway plays a major role in either process. Normal activation of the PI3K-AKT signaling pathway is basal for maintaining the anti-apoptotic pathway and possibly maintaining autophagy^95^. Overexpression of RAGE in the ovarian microenvironment may activate the PI3K-AKT-mTORC1 pathway in pregranulosa cells, reverse G0 to the G1 stage and increase the production and release of inflammatory cytokines, which also increases PI3K-AKT-mTORC1 activity in oocytes, leading to increased protein synthesis and eliminating the inhibition of growth by FOXO3a^96,97^. The mTOR signaling pathway exerts negative control over autophagy^98^. Under favorable conditions such as abundant nutrients and growth factors, mTOR is activated^99^. Activation of mTOR via the PI3K-Akt signaling pathway, mTOR phosphorylates serine-threonine kinase 1 (ULK1), a key kinase for the initiation of autophagy, thereby inhibiting the initiation of autophagy. Phosphorylated ULK1 is functionally inactivated during the early phase of autophagosome formation, disrupting the autophagic process^100^.

Beclin-1, a pivotal protein central to autophagosome nucleation, dynamically interacts with diverse regulatory molecules to orchestrate autophagy initiation^101^. Notably, Bcl-2 family proteins exert critical regulatory roles through direct binding to Beclin-1. As an anti-apoptotic protein, Bcl-2 sequesters Beclin-1 by forming a physical complex, thereby suppressing its pro-autophagic activity and inhibiting autophagosome formation^102^. This interaction occurs particularly at the endoplasmic reticulum and mitochondria under basal conditions. However, upon exposure to autophagy-inducing stimuli such as oxidative stress or nutrient deprivation, the Bcl-2-Beclin-1 association is disrupted. This dissociation liberates Beclin-1 to engage with class III PI3K, initiating the nucleation of isolation membranes critical for autophagosome biogenesis^103^. Conversely, when cells are subjected to autophagy-induced signals, such as certain stress signals, the binding of Bcl-2 to Beclin-1 dissociates, releasing the activity of Beclin-1 and promoting the formation of autophagosomes. In oocytes, this regulatory axis ensures quality control by enabling timely removal of aged mitochondria and oxidatively damaged organelles through autophagic flux^104^. However, excessive accumulation of AGEs and subsequent RAGE receptor activation dysregulates this balance. By activating the PI3K-AKT signaling pathway, this pro-oxidative stress condition disrupts Bcl-2-Beclin-1 binding. For instance, stress-activated kinases such as JNK or PKC may phosphorylate Bcl-2, reducing its affinity for Beclin-1. This phosphorylation event releases Beclin-1 to form functional complexes with Vps34 (class III PI3K), thereby promoting autophagosome assembly and the clearance of cytotoxic oxidative damage products^105^. This dual regulatory mechanism highlights the intimate crosstalk between apoptosis and autophagy mediated by the Bcl-2–Beclin-1 axis.

Other than that, RAGE may also be involved in the regulation of other physiological functions in granulosa cells. AGEs-RAGE interaction activates the MAPK-ERK signaling pathway, reducing the LH and FSH-induced ERK1/2 activation^106^. Inappropriate activation of ERK1/2 in granulosa cells may block the granulosa cell differentiation pathway and/or impair follicular responses to hormones, potentially leading to ovulation failure that characterizes polycystic ovarian syndrome^73^.

In ovarian granulosa cells under aging or pathological conditions, the high expression of RAGE may induce oxidative stress and impair autophagy, leading to granulosa cell dysfunction. Pathways such as the PI3K-AKT, MAPK-ERK, MAPK-JNK and NF-κB pathways are likely to mediate the adverse effects of RAGE on granulosa cells. However, the dominant pathway responsible for these effects remains unclear. Elucidating the roles of RAGE-mediated inflammatory signaling in granulosa cell dysfunction and its contribution to reproductive disorders will facilitate the development of more precise and effective clinical therapeutics targeting RAGE. Moreover, investigating the endocytic capacity of granulosa cells, exploring the coregulatory mechanisms of autophagy and apoptosis, and expanding the RAGE receptor expression profile could enhance our understanding of how granulosa cells influence oocyte survival and follicular atresia, as well as the cell type-specific responses to RAGE activation. Addressing these questions will provide insight into the biological role of RAGE in granulosa cells and novel therapeutic strategies for ovarian diseases.

### RAGE drives suppression of steroid synthesis and gonadotropin resistance in aging ovaries

The ovarian endocrine system primarily synthesizes steroid hormones, including estrogen and progesterone, through coordinated biosynthetic pathways in theca cells and granulosa cells^107^. Immunohistochemical analyses revealed RAGE localization in multiple ovarian cell populations, notably granulosa cells, theca interna cells and luteal cells, suggesting its regulatory potential in steroidogenic processes^55,91^. AGE–RAGE impairs Ca^2+^ mobilization and sensitization in colonic smooth muscle cells via the CAMP-PKA pathway^108^; thus, RAGE activation may impair progesterone biosynthesis via the suppression of the cAMP-PKA signaling axis, leading to the transcriptional downregulation of steroidogenic acute regulatory protein (StAR). This molecular perturbation reduces cholesterol transport into mitochondria, thereby limiting pregnenolone synthesis and subsequent progesterone production^109^. The pathophysiological effects of these receptors include oxidative stress modulation, where the RAGE–AGE interaction triggers NADPH oxidase activation, generating excessive ROS that induce mitochondrial dysfunction and compromise steroidogenic enzyme activities^110^. Notably, RAGE activation may induce gonadotropin resistance through inflammatory crosstalk. Inflammatory cytokines, including IL-6 and TNF-α, activate the RAGE-MAPK signaling axis, which disrupts gonadotropin receptor signaling pathways and impairs FSH-stimulated aromatase activity, consequently diminishing granulosa cell responsiveness to gonadotropic stimulation^111^. Furthermore, RAGE overexpression may perturb progesterone (P4) secretion dynamics through two mechanisms: direct inhibition of FSH-mediated proestrogenic signaling in granulosa cells and indirect impairment of luteal function via dysregulation of LH receptor signaling^112,113^.

In summary, direct evidence demonstrating the impact of RAGE on the production and function of ovarian hormones is still lacking, and further clinical data and experimental studies for validation are needed. We predict that RAGE may regulate ovarian hormones through multiple pathways. Future investigations should transcend the limitations of studying singular mechanisms by integrating dynamic monitoring, cross-omics analyses, and clinical cohort data. This approach will facilitate the translation of RAGE-related research from fundamental mechanisms to precision therapies, ultimately providing innovative intervention strategies for ovarian endocrine disorders.

### RAGE-mediated ovarian fibrosis via inflammatory-oxidative pathways

In ovarian aging, the overexpression of RAGE may induce ovarian fibrosis through activation of the inflammatory-oxidative stress network and pro-fibrotic signaling pathways, ultimately leading to excessive ECM deposition within the ovarian stroma. This pathological process potentially accelerates ovarian functional decline, manifested as reduced follicular reserve, hormonal dysregulation and tissue sclerosis^114^.

With advancing age or metabolic disorders, AGEs occurs systemically. Within ovarian tissue, AGEs bind to RAGE and activate downstream signaling cascades (including NADPH oxidase, MAPK and NF-κB pathways), triggering inflammatory responses and oxidative stress^24^. Subsequent RAGE activation promotes nuclear translocation of transcription factors such as NF-κB, which upregulates proinflammatory cytokines including TNF-α and IL-6, establishing a state of chronic low-grade inflammation. Persistent inflammatory and oxidative microenvironments activate ovarian stromal fibroblasts, initiating fibrotic repair mechanisms. These fibroblasts undergo differentiation into myofibroblasts that secrete excessive collagen and ECM components, ultimately resulting in ovarian tissue sclerosis and fibrosis^115^.

In addition, RAGE may exacerbate fibrotic pathological changes by amplifying the pro-fibrotic effects of TGF-β, thereby accelerating ECM deposition. In diabetic nephropathy, AGE–RAGE-mediated ROS generation activates the TGF-β–Smad signaling pathway, which subsequently induces mesangial cell hypertrophy and fibronectin synthesis via autocrine production of angiotensin II^116^. Furthermore, in endometrial cancer, RAGE has been shown to promote fibrosis in human endometrial stromal cells through activation of the JAK2–STAT3 signaling pathway^117^.

RAGE overexpression may also impair ovarian vascular function, inducing local ischemia-hypoxia that further potentiates fibrogenesis. Under physiological conditions, AGE–RAGE interactions reduce MMP-mediated ECM degradation and impair ischemia-induced angiogenesis^118^. Paradoxically, pathological conditions such as diabetes and atherosclerosis demonstrate RAGE-mediated promotion of aberrant angiogenesis. AGE–RAGE interactions accelerate vascular inflammation and plaque formation, enhance vascular smooth muscle cell proliferation/migration and promote neointimal hyperplasia, ultimately increasing vascular instability and thrombotic risk^119^.

**Fig. 4:**
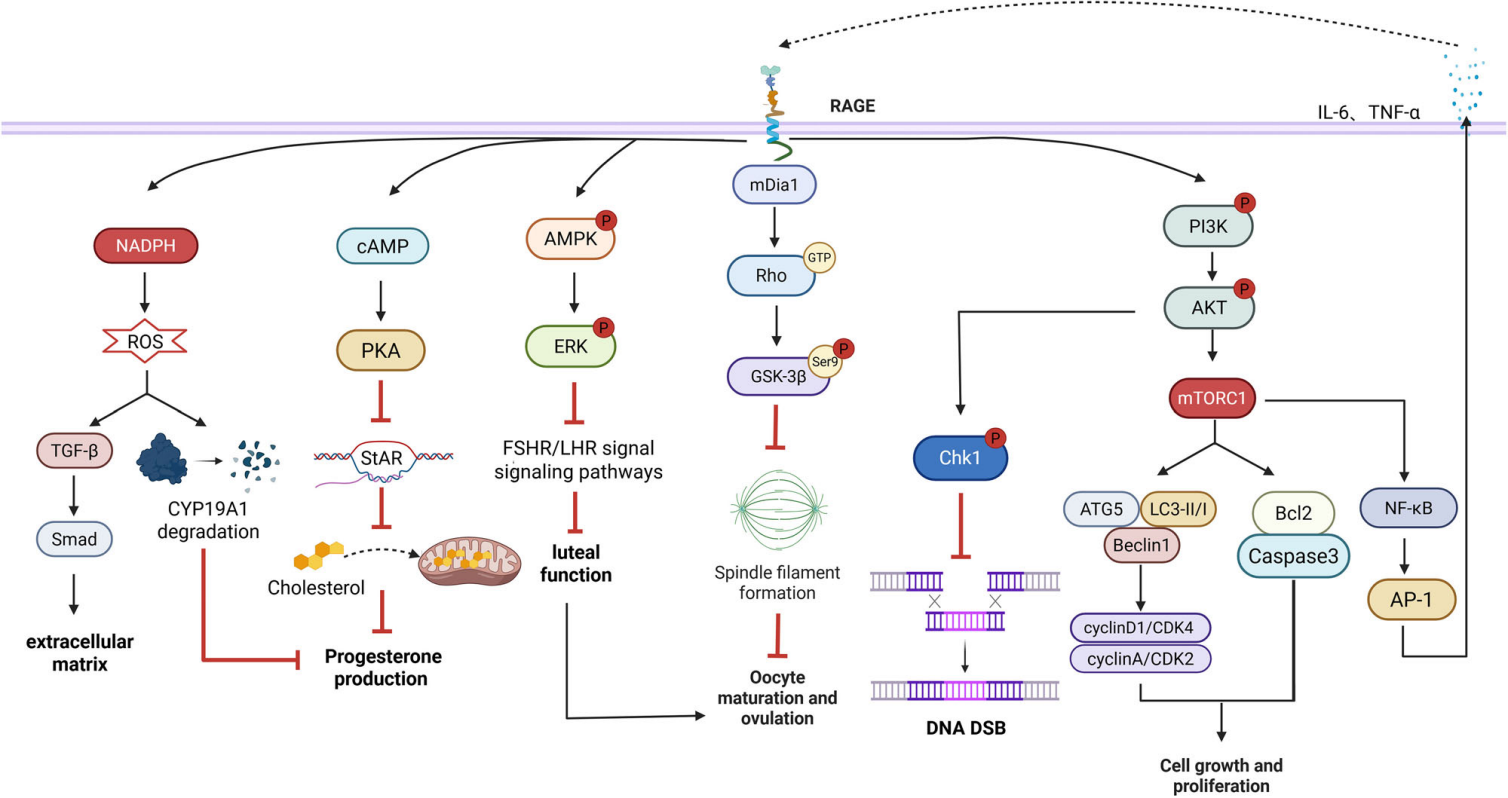
Schematic diagram of the mechanism by which RAGE affects ovarian function. The binding of RAGE to ligands in the ovaries may disrupt oocyte development, granulosa cell function and sex hormone synthesis through a variety of signaling pathways. Image created with BioRender; https://www.biorender.com/.

Furthermore, ovarian aging models and POF exhibit enhanced collagen deposition and upregulation of fibrotic markers (α-SMA and fibronectin), which show positive correlation with RAGE signaling activation levels. These findings collectively suggest RAGE serves as a critical molecular nexus connecting metabolic dysregulation, inflammatory cascades and fibrotic remodeling in ovarian senescence^120^ (Fig. 4).

## RAGE and the ovarian pathological process

The abnormal overexpression of RAGE has been established as a pivotal nexus linking metabolic stress, chronic inflammation and tissue senescence^78,121^. Within the ovarian microenvironment, persistent stimuli such as metabolic disturbances and oxidative stress can lead to the hyperactivation of RAGE signaling pathways^122^. This process not only directly drives ovarian biological aging by inducing granulosa cell dysfunction, accelerating follicular depletion and promoting ovarian stromal fibrosis^123,124^ but also constitutes a common pathological underpinning for the development of various ovarian disorders^125^. Downstream events mediated by RAGE, including chronic low-grade inflammation, excessive autophagy and apoptosis, act as a ‘molecular bridge’ that intimately connects the process of ovarian aging to a spectrum of functional and organic pathologies^126^. The following sections will elaborate on the specific expression patterns and molecular mechanisms of RAGE in major ovarian diseases such as premature ovarian insufficiency (POI), PCOS and ovarian cancer. This discussion will systematically explain how RAGE contributes to and modulates the pathogenesis and progression of these diseases by promoting ovarian aging (Table 1).

**Table 1:** Effects of RAGE studied in the various ovarian diseases.

Ovarian disease	Cells involved	Roles	Pathological process	Proteins/pathways involved	References
POF	Oocytes, ovarian granulosa cells	(1) Promote oxidative stress	(1) Mitochondrial dysfunction; (2) decreased estrogen and progesterone production	(1) NOX2/4↑ → ROS↑ → mtDNA↓ → ATP↓; (2) ROS↑ → transport of steroidogenesis substrates↓	^132–134^
		(2) Sustain chronic inflammatory responses	Aberrant activation and depletion of primordial follicles	NF-κB/MAPK↑ → IL-6/TNF-α/IL-1β↑	^135,138,139^
		(3) Dysregulation of apoptosis and autophagy	Damaged organelles clear obstacles and the intracellular accumulation of cytotoxic substances	(1) ROS↑ → Bax/Bak↑ → cytochrome C → caspase-9/3 cascade; (2) Fas/FasL↑ → DISC↑ → tBid/caspase-8↑; (2) mTOR↑ → Beclin-1 and LC3-II↑	^63,104,140–142^
PCOS	Ovarian granulosa cells, ovarian stromal cells, adipocytes, pancreatic β cell	(1) Promote androgen synthesis	Hyperandrogenemia	Aromatase↓ → estradiol↓; CYP17A1↑ → androgen↑	^112,152,160^
		(2) Impairing pancreatic β cell function	Insulin resistance and hyperinsulinemia	Phosphorylation of IRSs↓ → PI3K/Akt ↓	^153–155^
		(3) Sustain chronic inflammatory responses	Anovulation and polycystic changes in the ovaries	DAMPs↑ → macrophage (M1)↑ → IL-6/TNF-α/IL-1β↑	^56,163^
Ovarian cancer	Ovarian cancer cell, OSCs, neutrophils, mesenchymal cells	(1) Activation of an autocrine signaling loop	Cancer cell migration	mDia1↑ → Rho GTPases↑ → phosphorylation of GSK-3β/ROCK↑	^38,169–171,173^
		(2) Limit cancer cell apoptosis	Tumor growth	p53-dependent mitochondrial pathway/PI3K/AKT	^174^
		(3) OSCs undergo epithelial to mesenchymal transitions	Ovarian fibrosis	TGF-β↑	^175^

### POF

POF, a reproductive endocrine disorder characterized by a decrease in ovarian function before the age of 40, clinically manifests as amenorrhea, infertility, hypoestrogenemia and elevated gonadotropin levels^127,128^. Its etiology involves a complex interplay of multifactorial interactions, including genetic variants, autoimmune dysregulation, oxidative stress, and environmental toxins. Recent studies have highlighted the emerging role of the receptor for RAGE in ovarian aging. As a pattern recognition receptor, RAGE accelerates follicular depletion and functional decline in granulosa cells by mediating chronic inflammatory responses, oxidative damage and apoptotic signaling pathways.

Substantial clinical evidence indicates an association between RAGE and POF. Metabolic disorders such as diabetes and obesity are associated with a markedly greater incidence of POF, a phenomenon mechanistically linked to elevated circulating AGE levels and overactivation of ovarian RAGE signaling^129^. Hyperglycemic environments induce granulosa cell insulin resistance through the AGE–RAGE–ROS axis, inhibit FSH-mediated estrogen synthesis and promote follicular atresia^130,131^.

Current evidence suggests that key pathological mechanisms of RAGE-mediated POF involve the following pathways:

1. Oxidative stress and mitochondrial dysfunction: in addition to RAGE binding to AGEs, the activation of NADPH oxidases (NOX2/4) triggers a surge in ROS^132^. Excessive ROS not only directly damage mtDNA but also suppress the activity of electron transport chain complexes, leading to reduced ATP synthesis and mitochondrial membrane potential collapse^133^. In granulosa cells, mitochondrial dysfunction further disrupts the transport of steroidogenesis substrates, impairing the biosynthesis of estrogen and progesterone^134^. RAGE inhibition may increase antioxidant enzyme activities in ovarian tissue, reduce follicular atresia and preserve the ovarian reserve.
2. In the inflammatory signaling cascade and imbalance in the follicular microenvironment, RAGE, a proinflammatory receptor, drives excessive secretion of inflammatory cytokines (IL-6, TNF-α and IL-1β) via the NF-κB and MAPK pathways^135^. These cytokines establish a positive feedback loop that further activates RAGE and promotes M1 macrophage infiltration, compromising follicular wall integrity^136,137^. Clinical studies have revealed a positive correlation between IL-6 levels and RAGE expression in the follicular fluid of patients with POI, with hyperinflammatory states accelerating aberrant activation and depletion of primordial follicles^138,139^.
3. Dysregulation of apoptosis and autophagy: RAGE signaling elicits granulosa cell apoptosis through multiple pathways. RAGE activation leads to mitochondrial oxidative stress and the ROS generated within mitochondria activate Bax-Bak proteins, triggering mitochondrial cytochrome C release and subsequent activation of the caspase-9/3 cascade^140^. Furthermore, RAGE synergizes with Fas-FasL signaling to potentiate the extrinsic apoptotic pathway mediated by caspase-8. RAGE overexpression upregulates the expression of Fas (CD95) and its ligand FasL and promotes the formation of a death-inducing signaling complex (DISC), which recruits and activates caspase-8, directly cleaves caspase-3, or amplifies the apoptotic signal of the mitochondrial pathway via the Bid protein (tBid)^85,141^. Concurrently, RAGE overexpression may suppress the expression of critical autophagic proteins (Beclin-1 and LC3-II), impairing the clearance of damaged organelles and exacerbating the intracellular accumulation of cytotoxic substances^1,142^. However, RAGE signaling may trigger autophagic cell death through overactivation of autophagy (inhibition via the ROS-mTOR pathway). The autophagy-related protein Beclin-1 dissociates from Bcl-2 and promotes autophagosome formation while releasing proapoptotic factors^143^.

As the core molecule connecting metabolic disorders, oxidative stress and ovarian aging, RAGE plays a key role in the occurrence and development of POF. Targeting the RAGE signaling pathway provides a new idea for restoring ovarian function, but its clinical translation still needs to solve the problems of species differences, biomarker optimization and long-term safety evaluation. Future research should integrate multiomics technology and organoid models to analyze the dynamic regulation of the RAGE network in the ovarian microenvironment in detail and promote the development of individualized treatment options.

### PCOS

PCOS is one of the most common reproductive endocrine and metabolic disorders in women of childbearing age, with a global incidence of approximately 5–20%^144^. Clinical features include hyperandrogenism, oligoanitrusion or anovulation, and polycystic changes in the ovaries, which are often associated with insulin resistance, obesity, chronic inflammation and metabolic syndrome^11^.

The intrinsic link between PCOS and ovarian aging is increasingly being elucidated as a pathological process centered on locally ‘accelerated aging’ within the ovary. The core mechanism lies in the hyperandrogenic microenvironment within the ovaries of patients with PCOS, which directly induces premature cellular senescence in granulosa cells, manifested by the overexpression of senescence markers such as p16^145–148^. The dysfunction of these senescent granulosa cells leads to follicular development disorder and ovulation failure, representing a direct cause of diminished fertility^149^. Concurrently, these senescent cells persistently secrete a large quantity of inflammatory factors, chemokines and others, forming the senescence-associated secretory phenotype (SASP)^147^. This creates a chronic, low-grade inflammatory microenvironment within the ovary, which not only further impairs follicular health but may also exacerbate PCOS-related metabolic disorders through systemic effects. Furthermore, the AGE–RAGE signaling axis serves as a crucial metabolic and inflammatory nexus, linking the metabolic abnormalities common in PCOS to ovarian aging^150^: the binding of high levels of AGEs to their receptor (RAGE) can aggravate oxidative stress and inflammatory responses, thereby accelerating the senescence process of ovarian cells^78,151^. This series of chain reactions—from endocrine disturbance (hyperandrogenism) to cellular events (granulosa cell senescence), to microenvironmental alteration (SASP-induced inflammation) and metabolic interaction (AGE–RAGE axis)—collectively constitutes the pathological basis for the premature decline of ovarian function in patients with PCOS. Therefore, exploring the association between RAGE and PCOS and its potential molecular mechanism has gradually become a hot topic in the study of the pathological mechanism of PCOS.

The clinical manifestation of hyperandrogenemia in patients with PCOS may be associated with high expression of the RAGE protein. RAGE signaling may reduce the conversion of testosterone to estradiol by inhibiting aromatase activity in ovarian granulosa cells and upregulate the expression of CYP17A1 in ovarian stromal cells, promoting androgen synthesis. The excessive accumulation of AGEs in patients with PCOS is closely related to the increase in the glycation response in a high-glucose environment, resulting in overactivation of RAGE receptors. RAGE receptor activation causes inflammation, apoptosis and oxidative stress by activating multiple signaling pathways, including the NF-κB and MAPK pathways, thereby exacerbating insulin resistance, impairing pancreatic β cell function and dysregulating glucose metabolism^152^. RAGE plays a dual role in insulin signaling pathways: on the one hand, AGE–RAGE activation suppresses tyrosine phosphorylation of insulin receptor substrates (IRSs), impairing PI3K-Akt signaling^153–155^; on the other hand, RAGE exacerbates insulin resistance in the liver and skeletal muscle by inducing endoplasmic reticulum stress and mitochondrial dysfunction^156,157^. Clinical intervention trials have demonstrated that metformin not only improves insulin sensitivity in PCOS patients but also reduces the level of serum AGEs and the protein expression of RAGE^158^.

The specific mechanisms of RAGE activation in PCOS may depend on a hyperglycemic environment and the accumulation of AGEs. Patients with PCOS often present with insulin resistance and hyperinsulinemia, leading to elevated circulating glucose levels^159^. Chronic hyperglycemia promotes AGE formation through nonenzymatic glycation reactions that modify proteins, resulting in the formation of irreversible AGE–RAGE complexes. Studies have revealed that serum AGE levels are markedly higher in patients with PCOS than in healthy women and are positively correlated with hyperandrogenemia and ovarian dysfunction^27^. Following AGE–RAGE axis activation, the NF-κB signaling pathway in ovarian granulosa cells is triggered, which suppresses the expression of steroidogenic enzymes and results in reduced estrogen synthesis and excessive androgen secretion^112,160^. The oxidative stress state in PCOS patients has been well documented, with high oxidative stress levels further amplifying RAGE signaling. Mitochondrial dysfunction and NADPH oxidase activation lead to excessive ROS production, which activates RAGE and establishes a ‘ROS–RAGE’ positive feedback loop, suggesting that RAGE may contribute to ovarian damage in PCOS by regulating oxidative stress^161,162^. In obese patients with PCOS, RAGE expression is markedly elevated in adipose tissue. DAMPs such as HMGB1 and S100A8/A9 secreted by adipocytes bind to RAGE, promoting macrophage polarization toward the M1 phenotype and releasing proinflammatory cytokines, thereby exacerbating systemic low-grade inflammation^56,163^.

Research has confirmed that RAGE plays a substantial role in the onset and progression of PCOS by mediating inflammation, oxidative stress and metabolic disturbances. However, the following issues still require further exploration: (1) the heterogeneity of RAGE signaling across different PCOS phenotypes (such as obese versus nonobese); (2) the interplay between RAGE and the gut microbiota, as well as epigenetic regulation; and (3) the long-term safety of targeted RAGE therapy and its impact on reproductive outcomes. Future studies should integrate multiomics technologies and clinical translation to identify new targets for the precise intervention of PCOS.

### Ovarian cancer

Ovarian cancer is one of the most lethal malignancies in gynecology, with approximately 70% of patients being diagnosed at an advanced stage (III/IV), and the 5-year survival rate is less than 30%^164^. Its high invasiveness and propensity for recurrence are closely related to the complex regulation of the tumor microenvironment^165^. The high recurrence rate and resistance to chemotherapy have prompted researchers to explore new molecular targets. RAGE, a key regulator of proinflammatory and procancer signaling, has been shown to be associated with tumor progression in various solid tumors^166^. In recent years, RAGE has been found to play crucial roles in the development, metastasis and drug resistance of ovarian cancer.

Analysis of clinical samples revealed that the expression of RAGE in ovarian cancer tissues is markedly higher than in normal ovarian epithelial tissues, and it is positively correlated with FIGO stage, lymph node metastasis and chemotherapy resistance^29^. Some genetic variants in the RAGE gene can alter RAGE expression and function, thereby influencing the development of the disease^167^. The RAGE 82G>S variant was strongly associated with the risk of epithelial ovarian cancer in the Chinese cohort. The risk of epithelial ovarian cancer in 82SS genotype carriers was 2.65× greater than that in 82GG genotype carriers. These results suggest that the 82G>S polymorphism of the RAGE gene can be used as a genetic marker for epithelial ovarian cancer^168^. In approximately 60% of serous ovarian cancer cases, RAGE expression is upregulated, and the colocalization of RAGE with HMGB1 in clear cell carcinoma suggests the activation of an autocrine signaling loop^169^. The cytoplasmic domain of RAGE binds to the form of mammalian diaphanous-1 (mDia1); in transformed cells, macrophages and SMCs, mDia1 is essential for RAGE ligand (AGE and S100B)-mediated activation of Rho GTPases and downstream signal transduction, lamellipodia formation and cellular migration^38,170,171^. Rai et al. inhibited the engraftment and metastasis of an lysophosphatidic acid (LPA)-induced epithelial ovarian cancer cell line (ID8) and inhibited the occurrence of ovarian cancer by using sRAGE or RAGE knockout^172^, suggesting that migration induced by S100B-RAGE signaling via mDia1 requires serine 9 phosphorylation of GSK-3β^171^ and the ROCK signaling pathway^173^.

There are many explanations for the involvement of RAGE in the pathogenesis of ovarian cancer. RAGE can limit apoptosis through the p53-dependent mitochondrial pathway and directly links inflammatory mediators in the tumor microenvironment to apoptosis resistance^174^. Functional RAGE is present on the plasma membrane of human neutrophils, and RAGE involvement impairs neutrophil function; thus, RAGE acts as an important mediator of oogonia stem cells (OSC) damage to interfere with antitumour immunity, promote angiogenesis and support tumor growth^168^. On the other hand, RAGE-mediated TGF-β secretion can also promote the growth of mesenchymal cells, and OSC cells undergo epithelial to mesenchymal transitions, resulting in the growth of higher-grade OSCs^175^. The activation of the RAGE-PI3K-AKT signaling pathway may be involved in SKOV3 ovarian carcinogenesis, chemoresistance and metastasis^176^. RAGE is also inextricably linked to the apoptosis of ovarian cancer cells. RAGEs binding to acetylated APE1-Ref-1 promoted the apoptosis of ovarian cancer cells. An animal model revealed that RAGE knockdown reduced cell death and poly(ADP‒ribose) polymerase cleavage caused by rhAPE1-Ref-1 and aspirin (ASA) combination treatment, highlighting the importance of the APE1-Ref-1‒RAGE interaction in triggering apoptosis^177^.

As a key regulator of ovarian cancer progression, the multiligand characteristics and complex signaling network of RAGE provide new ideas for treatment, but the development of new methods for the treatment of ovarian cancer for RAEG still faces many problems: (1) the differences in RAGE signaling pathways in ovarian cancer subtypes (such as high-grade serous carcinoma and mucinous carcinoma) need to be studied in detail; (2) targeting specificity: the physiological function of RAGE in normal tissues (the lungs and brain) may cause off-target toxicity; (3) the nanoparticle drug delivery system is expected to improve the enrichment of RAGE inhibitors in peritoneal metastases. In the future, combining multiomics technology and clinical translational research to optimize targeting strategies and explore individualized treatment options is necessary.

## The potential of RAGE as a therapeutic target for ovarian aging

**Fig. 5:**
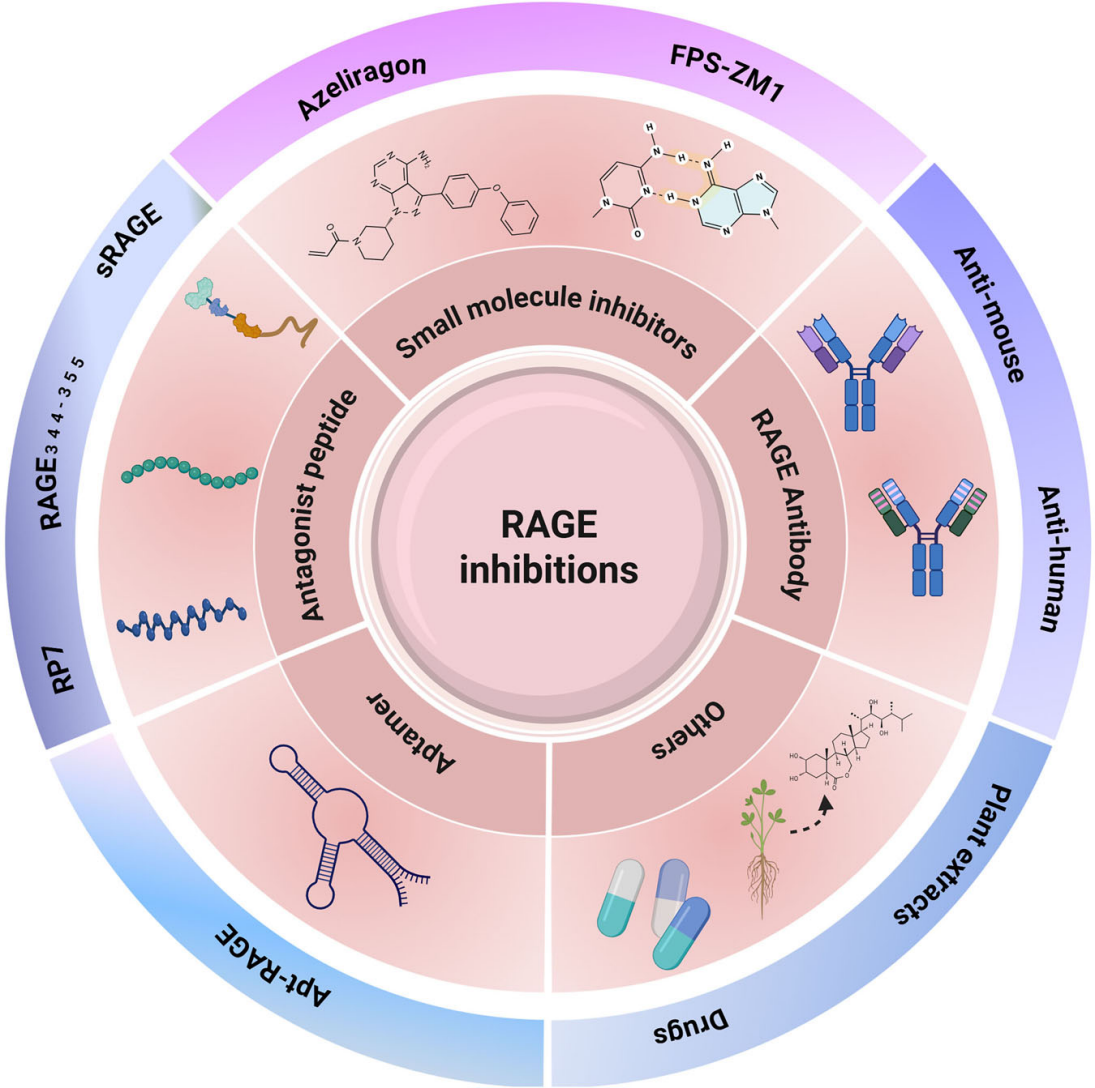
RAGE inhibitors classification. RAGE inhibitors primarily encompass small molecule inhibitors, monoclonal antibodies, antagonistic peptides, aptamers, natural products and repurposed drugs with established clinical applications in other disease contexts. Image created with BioRender; https://www.biorender.com/.

Ovarian aging is the core marker of deterioration of female reproductive system function and manifests as a decrease in the number of follicles, an imbalance in hormone secretion and a decrease in fertility, and its mechanisms involve oxidative stress, chronic inflammation and mitochondrial dysfunction. As a key regulator of proinflammatory and oxidative damage, RAGE is closely related to the process of ovarian aging and has become a potential intervention target for delaying reproductive aging. A previous study revealed that RAGE-knockout mice are healthy and normally develop, suggesting that RAGE knockdown may be a safe therapeutic strategy^178^. In recent years, a number of extracellular ligand-based RAGE inhibitors have also been shown to be effective in treating RAGE-mediated diseases (Fig. 5 and Table 2).

**Table 2:** Therapeutic strategies targeting RAGE.

Intervention	Classification	Compounds	Effect and mechanism	References
RAGE inhibition	Small molecule inhibitors	Azeliragon (TTP488)	JAK1↓ → STAT3↓ → NF-κB↓ → IRF3↓ → NLRP3↓ → proinflammatory cytokines↓	^179^
		FPS-ZM1	Blocking the binding of ligands such as AGEs, HMGB1 and S100A8/A9 to the RAGE protein	^196^
		Matrine	Blocking the RAGE–Aβ axis	^207^
		Thiazole derivative 22n	Blocking Aβ–RAGE binding to inhibite the transport of Aβ across the BBB and NF-κB activation	^208^
		4,6-Disubstituted 2-aminopyrimidines (4,6-bis(4-chlorophenyl)pyrimidine (compound 59); 4-fluorophenoxy analog (40); 3-(*N*,*N*-dimethylamino)pyrrolidine analog 12o)	Inhibiting RAGE–Aβ interactions to reduce toxic soluble Aβ levels in the brain and improve cognitive function	^209–211^
		Compound 48	Suppressing Aβ-induced toxicity in mouse hippocampal neuronal cells	^212^
	Antibody	Anti-human RAGE monoclonal antibodies (MAB1145 (R&D Systems); ab216329 (Abcam); sc-365154 (Santa Cruz Biotechnology); CR-3)	Blockading RAGE activation or expression and preventing downstream signaling	^214,215^
		Anti-mouse RAGE antibodies [MAB1179 (R&D Systems); AF1179 (R&D Systems)]	Blockading RAGE activation or expression and preventing downstream signaling	^213^
		Human‒mouse chimeric antibody A5 (HuA5)	NF-κB↓	^216^
	Peptide	RP7	Phosphorylation of ERK1/2, IKKα/β, IKBα and p65↓ → Bcl-2/HMGB1↓ → apoptosis↑ → EMT↓	^218,219^
		RAGE_344-355_	eEF1a1 → ROS↓	^57^
		sRAGE	p-ERK↓ → AP-1↓	
	Aptamer	AptRAGE	Against the AGE–RAGE axis	^229^
	Natural agents	Erxian decoction	RAGE↓ → PI3K↓ → proapoptotic proteins (BAX and CASPASE3)↓ → senescence markers (p16, p21, p53 and lamin A/C)↓ → BCL-2↑	^237^
		Acacetin	Attenuating LPA-mediated RAGE-PI3K/AKT signaling activation	^176^
		Naringin	Ameliorating type 2 diabetes-associated steatohepatitis via the suppression of RAGE/NF-κB-mediated mitochondrial apoptosis pathways	^238^
		Geniposide	Suppressing inflammatory cascades in diabetic nephropathy models	^239^
	Drugs	Adiponectin	Reducing SASP-related proinflammatory cytokines (IL-6 and IL-8), preserve telomeric integrity and decrease SA-β-gal accumulation	^240^
		Metformin	PI3K↑ → Akt↑ → FOXO3a↑ → CASPASE3↓	^28^
		1,25-Dihydroxyvitamin D3	Suppressing of steroidogenic gene expression	^160^
		Coenzyme Q10	Reducing postprandial oxidative stress and serum AGE levels	^241^
Combination therapies	RAGE monoclonal antibody and TLR4 inhibitor	–	Preventing post-traumatic epilepsy	^242^
	RAGE inhibitor and PD-1 inhibitor	–	Eliminating postoperative cerebral edema	^243^

### Small molecule inhibitors of RAGE

#### Azeliragon

Azeliragon, also known as TTP488 and PF-04494700, was developed by TransTech Pharma in the USA as an oral inhibitor of RAGE in Alzheimer’s disease. Azeliragon is the first orally bioavailable small-molecule inhibitor of RAGE that works by inhibiting RAGE. Azeliragon can block the interaction of RAGE–amyloid β peptide (Aβ) in patients with Alzheimer’s disease, thereby achieving a therapeutic effect on Alzheimer’s disease^179^. TTP448 can reverse the activation of the JAK1-STAT3-NF-κB-IRF3 pathway and the expression of NLRP3 induced by Aβ, reduce the expression of proinflammatory cytokines (interleukin (IL)-1β, IL-6 and TNF-α) in patients with Alzheimer’s disease, reverse the inhibitory effect of Aβ on cell proliferation and viability, and reduce apoptosis and ROS production^180^. The inhibitor is currently in phase 3 clinical development, but its potential benefits are inconclusive. Azeliragon has a good safety profile when patients take a low dose of 5 mg/day, slowing cognitive decline at 18 months. However, trials have shown that taking high doses of 20 mg/day is associated with cognitive decline. Patients treated at 30 mg/day for 10 weeks had a lower incidence of side effects, no clinically meaningful differences in vital signs and so on but no consistent effects on certain biomarkers or cognitive outcomes^181–183^.

In recent years, Azeliragon has been used to treat a variety of diseases, including uremia, diabetic neuropathy, acute lung injury, diabetic neuropathy and nonalcoholic steatohepatitis^184–187^, by inhibiting multiple RAGE-mediated signaling pathways. Azeliragon can also improve the rejection of T cells to xenografts, inhibit the metastasis of triple-negative breast cancer and improve glioblastoma microenvironment structure-related inflammation, among other effects^188–190^.

**Fig. 6:**
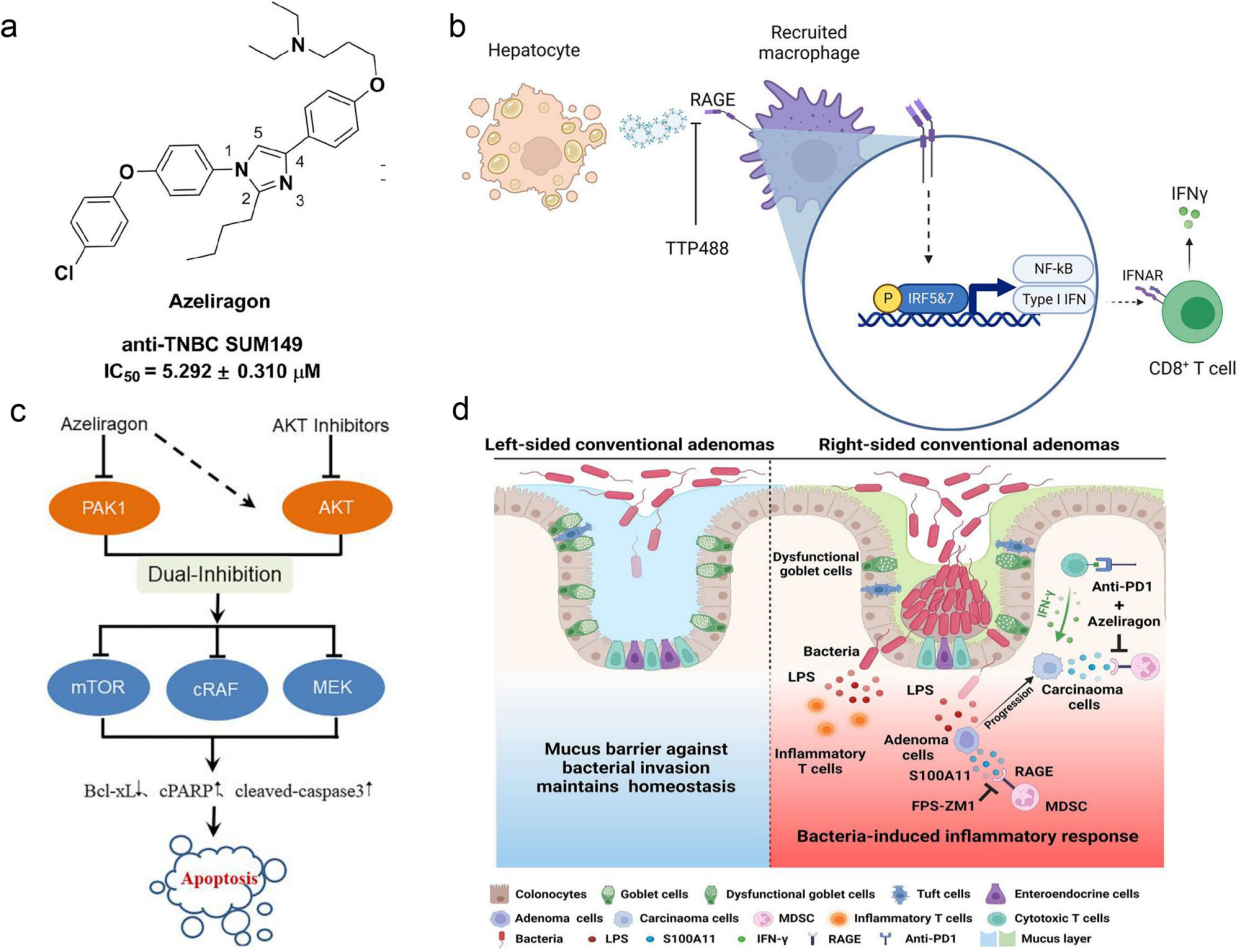
Structure and mechanisms of Azeliragon. **a** The structure of Azeliragon^195^. **b** RAGE+ macrophage–T cell crosstalk drives NASH inflammation^187^. **c** Azeliragon/AKT inhibitor synergy in pancreatic cancer^193^. **d** Azeliragon may aid immunotherapy via blocking S100A11-RAGE-mediated MDSC recruitment in colon adenomas^194^.

Azeliragon is also expected to be used in combination with other drugs to enhance the effect of the drug or exert multiple therapeutic effects. Tofacitinib and fludarabine administration can further reverse Alzheimer’s disease injury after TTP488 intervention^191^. The combination of vilazodone and azeliragon may treat the comorbidities of Alzheimer’s disease and depression with the potential to form a novel dual RAGE-SERT inhibitor^192^. Azeliragon can also enhance the therapeutic effect of AKT inhibitors in pancreatic cancer by inhibiting PAK1^193^. RAEG is expected to be a therapeutic target to increase anti-PD-1 efficacy, and Azeliragon can be combined with an anti-PD-L1 antibody to inhibit immune evasion from right-sided colonic adenoma and enhance antitumour effects^187,193–195^ (Fig. 6).

#### FPS-ZM1

FPS-ZM1, a 327-Da RAGE-targeted inhibitor identified through systematic screening of a 5,000-member small-molecule chemical library, has nanomolar-range binding affinity for the V-type immunoglobulin domain of RAGE. Its blood‒brain barrier (BBB) permeability enables central nervous system targeting, where it mechanistically attenuates Aβ-triggered cellular stress responses in RAGE-positive cells via selective disruption of the Aβ–RAGE signaling axis^196^. FPS-ZM1 exhibits no toxicity in mice and readily crosses the BBB. Subsequent studies have demonstrated its ability to block the binding of ligands such as AGEs, HMGB1 and S100A8/A9 to the RAGE protein while demonstrating therapeutic efficacy across multiple disease models^197–199^.

FPS-ZM1 suppresses breast cancer cell invasion and metastasis, inhibits primary tumor growth, attenuates tumor angiogenesis and inflammatory cell recruitment and most notably prevents pulmonary and hepatic metastases^200^. It markedly ameliorates BBB disruption, cerebral edema, motor dysfunction and neural fiber damage while downregulating the expression of RAGE, NF-κB (p65), IL-1β, IL-6, IL-8R, COX-2, iNOS and MMP-9^201^. Through RAGE blockade, FPS-ZM1 enhances diabetic osteogenesis in adipose-derived stem cells via DNA methylation and Wnt signaling pathway modulation^202^. This compound has anti-inflammatory effects on LPS-activated microglia through JAK-STAT signaling pathway inhibition^203^. As a RAGE antagonist, FPS-ZM1 improves survival rates in SARS-CoV-2-infected mice by suppressing RAGE-mediated signal transduction, thereby mitigating systemic inflammation and perivascular pathology^204^. Pharmacological inhibition of the HMGB1-RAGE axis reverses cisplatin-induced ROS accumulation, apoptotic activation and the inflammatory response. Specifically, FPS-ZM1 counteracts cisplatin ototoxicity through HMGB1-RAGE signaling pathway suppression^205^. Mechanistically, AGEs impair orthodontic force-induced osteogenesis in periodontal ligament stem cells via the KDM6B-Wnt autoregulatory loop, whereas FPS-ZM1 enhances orthodontic treatment efficacy in diabetic patients by targeting the AGE-RAGE pathway^206^.

In summary, FPS-ZM1 exerts multiple biological effects, including anti-inflammatory, antioxidant, antifibrotic and antitumour effects, by inhibiting downstream proinflammatory and profibrotic signaling pathways of RAGE, such as the NF-κB, JAK-STAT, MAPK and Wnt pathways. However, the clinical application of FPS-ZM1 faces numerous challenges and limitations. Although it can penetrate the BBB, its oral bioavailability, tissue-specific distribution and half-life have not been fully elucidated, potentially necessitating structural optimization or improvements in delivery systems. Current research is predominantly based on animal models and cellular experiments (mice and stem cells), with a lack of data on human safety and efficacy. It has not yet entered phase II/III clinical trials, and the dose‒response relationship and long-term toxicity remain to be validated. The upstream and downstream regulatory networks of the RAGE signaling pathway exhibit considerable variability across different diseases, necessitating the development of individualized dosing regimens.

**Fig. 7:**
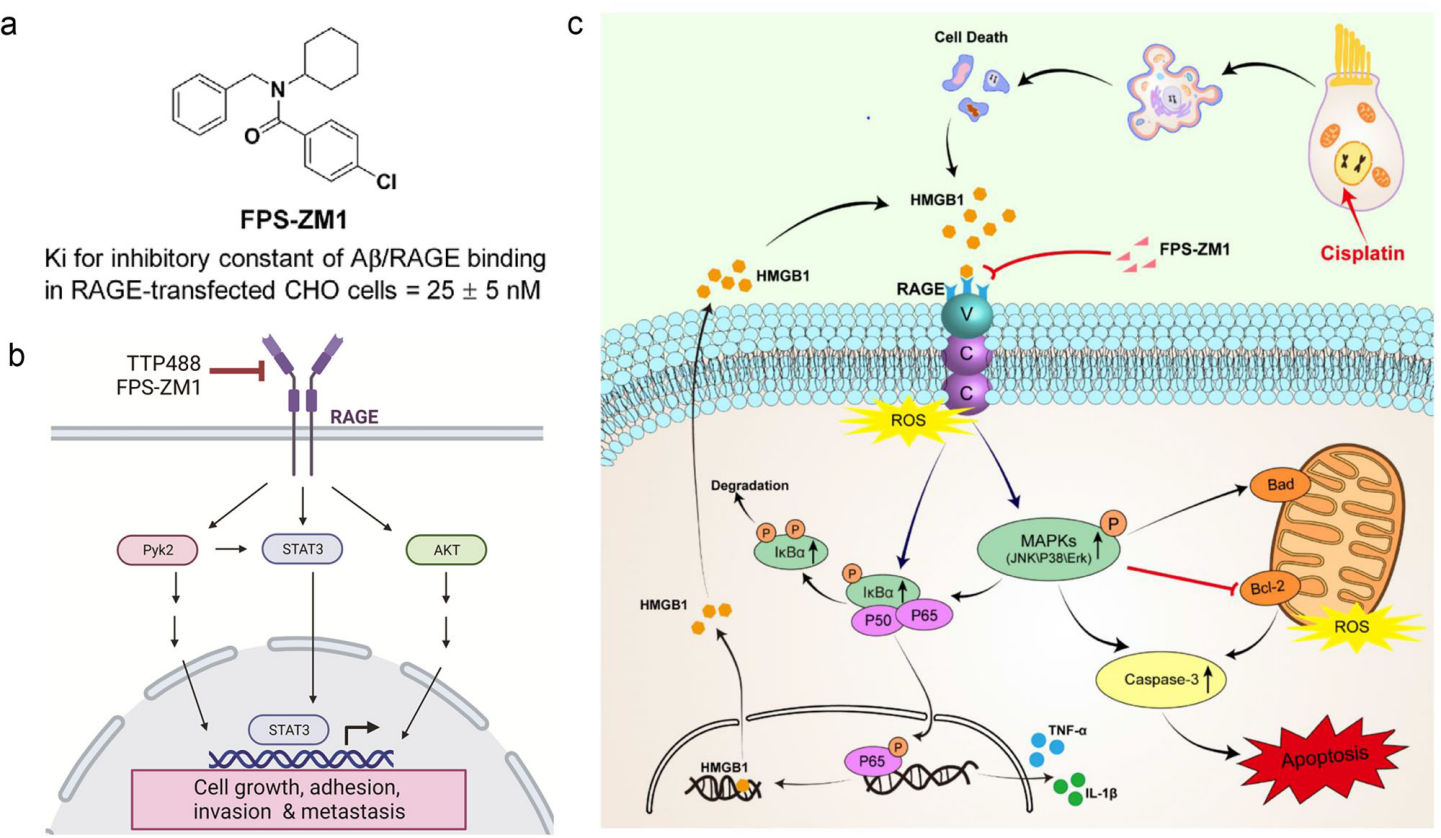
Structure and mechanisms of FPS-ZM1. **a** The structure of new high-affinity Aβ–RAGE blockers^196^. **b** RAGE signaling in TNBC drives tumor metastasis; a schematic depicting the major signaling pathways activated by RAGE in TNBC cells leading to metastasis^189^. **c** A schematic diagram of the possible beneficial mechanism of FPS-ZM1 in cochlear hair cells^205^.

As a specific inhibitor of the RAGE pathway, FPS-ZM1 has multidimensional therapeutic potential in the fields of oncology, metabolic diseases and inflammation. However, its clinical application still requires breakthroughs in pharmacokinetic optimization, enhanced targeting precision and large-scale clinical validation. Future research may focus on biomarker-guided precision therapy or the development of nanocarrier drug delivery systems to maximize efficacy and minimize risks^189,196,205^ (Fig. 7).

#### Other small molecule inhibitors of RAGE

The above two small-molecule inhibitors of RAGE are the most widely used in research. Azeliragon is the first RAGE inhibitor in a phase III clinical trial for the treatment of Alzheimer’s disease (NCT03980730). Unfortunately, the phase III clinical trial was terminated, possibly because of the poor safety of azeliragon^192^. Therefore, there is an urgent need to develop other efficacious RAGE blockers with high safety.

According to recent reports, a multitarget natural drug candidate, matrine, improves cognitive deficits in Alzheimer’s disease transgenic mice by inhibiting Aβ aggregation and blocking the RAGE–Aβ axis^207^. Similarly, the thiazole derivative 22n inhibited not only the transport of Aβ across the BBB but also Aβ-associated NF-κB activation by blocking Aβ–RAGE binding^208^. Building on this work, Han et al. employed ligand-based drug design to discover a novel series of 4,6-disubstituted 2-aminopyrimidines as RAGE inhibitors. Specifically, in transgenic Alzheimer’s disease mouse models, one analog—4,6-bis(4-chlorophenyl)pyrimidine (compound 59)—markedly reduced toxic soluble Aβ levels in the brain and improved cognitive function. Mechanistically, surface plasmon resonance analysis confirmed direct binding between compound 59 and RAGE, suggesting that its biological effects stem from the inhibition of RAGE–Aβ interactions^209^. The team subsequently analyzed the extensive structure‒activity relationship and identified a 4-fluorophenoxy analog (40) that exhibited improved in vitro RAGE inhibitory activity and more favorable aqueous solubility than the parent 2-aminopyrimidine. Surface plasmon resonance and molecular docking studies strongly supported the RAGE inhibitory activity of pyrazole-5-carboxamides^210^. By further refining their approach, they optimized the aminoalkoxy moiety of 4,6-bisphenyl-2-(3-alkoxyanilino)pyrimidine inhibitors, revealing that the tertiary amine group is critical for RAGE inhibition. This discovery led to the development of 3-(*N*,*N*-dimethylamino)pyrrolidine analog 12o, a promising RAGE inhibitor with superior activity and solubility. Molecular modeling further demonstrated that its enhanced efficacy arises from hydrogen bonding between the pyrrolidine nitrogen and Arg48, as well as hydrophobic interactions between the dimethylamino group and the RAGE binding site^211^. In parallel, Choi et al. reported several effective inhibitors that block RAGE–Aβ interactions without causing substantial cellular toxicity. Further testing revealed that compound 48 suppressed Aβ-induced toxicity in mouse hippocampal neuronal cells and reduced Aβ levels in the brains of a transgenic mouse model of Alzheimer’s disease after oral administration^212^.

**Fig. 8:**
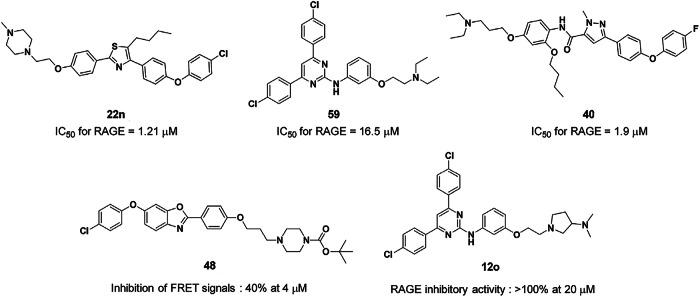
Reported anti-AD small molecule RAGE inhibitors^192^. Several small-molecule RAGE inhibitors (22n, 59, 40, 48, 12o, Matrine) have been identified as promising anti-Alzheimer’s disease agents. AD Alzheimer’s disease.

The development of small-molecule RAGE inhibitors represents a promising therapeutic strategy for multiple disease pathologies, particularly those involving chronic inflammation and protein‒protein interaction dysregulation. These compounds offer distinct pharmacological advantages over biologics, including superior oral bioavailability, enhanced metabolic stability and improved scalability for pharmaceutical manufacturing. Current research efforts focus on three critical fronts: (1) structure‒activity relationship optimizations to increase target specificity while preserving the native ligand-binding homeostasis of RAGE, (2) the development of broad-spectrum inhibitors capable of addressing the polymodal ligand recognition mechanism of RAGE and (3) a comprehensive assessment of pharmacokinetic profiles to ensure optimal BBB penetration where applicable. Key challenges in the field involve reconciling the receptor’s pleiotropic signaling pathways with selective inhibition strategies, particularly given the dual role of RAGE in both pathological inflammation and essential physiological processes^192^ (Fig. 8).

### Antibody-based drugs of RAGE

At present, no antibody drugs against RAGE have been approved for marketing, and more are in preclinical studies. Investigational RAGE antibodies are commonly used in in vitro studies (western blot and immunohistochemistry) or in animal models. Anti-human RAGE monoclonal antibodies include MAB1145 (R&D Systems), ab216329 (Abcam) and sc-365154 (Santa Cruz Biotechnology). Anti-mouse RAGE antibodies include MAB1179 (R&D Systems) and AF1179 (R&D Systems).

These antibodies have been used in a variety of studies. Antibodies developed by Abcam have shown that RAGE antibodies at doses of 10 and 100 ng/kg body weight improve diabetic retinopathy in Wistar rats through hypoglycemic effects and anti-inflammatory mechanisms^213^. The potential antiviral mechanism of RAGE antibodies was determined in SARS-CoV-2-infected human lung epithelial cells (BEAS-2B) in vitro. The results of preclinical studies revealed the antiviral and anti-inflammatory therapeutic effects of the RAGE-Ig protein on type I and type III IFN-mediated treatment of coronavirus disease 2019 caused by multiple SARS-CoV-2 variants of concern (VOCs)^214^. A humanized monoclonal anti-RAGE antibody (CR-3) improves hindlimb perfusion and angiogenesis in femoral artery (FA)-occlusive diabetic pigs. The contributing factors are increased collateral and decreased vascular RAGE expression^215^.

Chimeric antibodies have also been studied. Liu et al. fused the variable region of a mouse-derived antibody with the constant region of a human-derived antibody to construct the human‒mouse chimeric antibody A5, which targets RAGE. In addition, the humanized anti-RAGE chimeric antibody A5 was named HuA5 by site-directed mutagenesis, and it was verified that humanization did not change the affinity of the anti-RAGE antibody. These data show that blocking the RAGE pathway with huA5 strongly reduces liver injury and fibrosis. Anti-RAGE chimeric antibodies exert antifibrotic effects in vitro to reduce liver fibrosis in vivo. HuA5 could be further developed as a lead molecule for drugs to treat patients with liver fibrosis^216^.

In conclusion, RAGE antibody drugs are still in the research and development stage, and clinical translation is facing challenges. Research tool antibodies are well established to support mechanism exploration. Future research trends may lead to the optimization of efficacy through the engineering of antibodies, such as bispecific antibodies or nanobodies.

### RAGE antagonist peptide

At present, the global peptide drug market is growing rapidly. As of May 2024, there are more than 110 peptide-based drugs worldwide, with the market value expected to reach US$68.7 billion by the year 2030^217^. The field of oncology and inflammation is the core direction of peptide drugs, and the development of RAGE peptide inhibitors has also become a research hotspot.

Cai et al. screened eight novel RAGE-binding peptides with high binding affinity for RAGE via phage display technology^218^ and identified RP7 as the most capable of binding to the TNBC cell surface RAGE protein. RP7 inhibits tumor growth in TNBC xenograft mouse models by inhibiting the phosphorylation of ERK1/2, IKKα/β, IKBα and p65, blocking p65 from entering the nucleus and decreasing the protein expression of Bcl-2 and HMGB1, which activates TNBC cell apoptosis and inhibits epithelial‒mesenchymal transition (EMT) without inducing detectable toxicity in normal tissues^219^.

Proteomic analysis of human follicular fluid by Hou et al. revealed a novel peptide called RAGE_344-355_, which is cleaved from the RAGE protein and located at RAGE_344-355_ (ALGILGGLGTAA). This peptide can effectively improve oocyte development by interacting with eEF1a1 in oocytes to attenuate mitochondrial damage caused by oxidative stress in oocytes in mice with PCOS^57^.

**Fig. 9:**
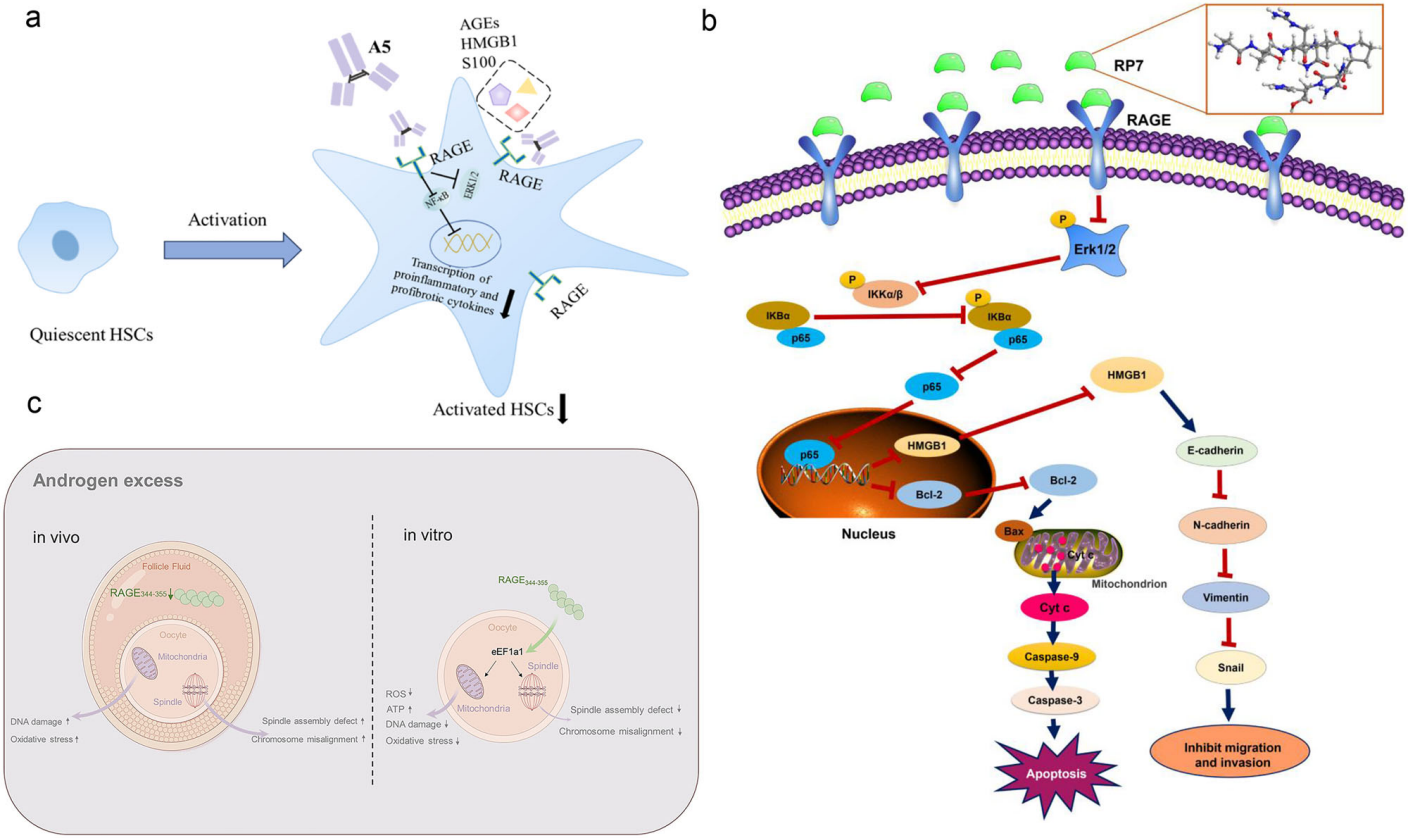
RAGE antibody and antagonist peptide. **a** A schematic representation of inhibition of hepatic stellate cell activation by anti-RAGE chimeric antibodies. A5 blocks ligands binding to RAGE, inhibiting its downstream NF-κB pathway and hepatic stellate cell activation^216^. **b** Schema showing a proposed mechanism of RP7. RP7 suppresses ERK1/2 phosphorylation, reducing NF-κB signaling, thereby promoting TNBC apoptosis via caspase activation and inhibiting metastasis by blocking HMGB1/EMT pathways^219^. **c** A diagram illustrating how experimental perturbations of RAGE_344-355_ affect PCOS mouse oocyte functions. RAGE_344-355_ supplementation in PCOS oocytes induces eEF1a1 to counteract ROS-mediated meiotic defects and oxidative stress^57^.

The use of transmembrane domain-deficient sRAGE as an endogenous RAGE antagonist was once considered a potential marker of the ovarian reserve and a therapeutic strategy for ovarian diseases. sRAGE can inhibit pathological signaling by competitively binding AGEs, treating ovarian-related diseases and delaying ovarian aging^220^. The immunohistochemical score of RAGE-AGEs in ovarian tissue is positively correlated with pathological grade and may be used to assess disease progression. sRAGE downregulates the expression of VEGF in ovarian hyperstimulation syndrome ovarian granulosa cells, which may involve the EGF-like growth factor pathway, and sRAGE may play a potential protective role in ovarian hyperstimulation syndrome ^203^. Exogenous sRAGE supplementation may alleviate inflammation in ovarian follicular granulosa cells by modulating p-ERK and AP-1 signaling^221^. The higher the FF sRAGE is, the lower the number of international units of Gn needed per cycle, suggesting that sRAGE may exert a protective effect on the follicular environment^57,216,219,222^ (Fig. 9).

### Aptamer

Aptamers consist of short single-stranded DNA or RNA sequences that bind to the target molecule and neutralize its function^223^. Protein antibodies are also widely used for the same purpose, but aptamers offer many advantages over neutralizing antibodies, such as short generation times, low production costs, low product-to-product variability and high thermal stability^224,225^. Currently, several clinical trials are underway to evaluate the efficacy of aptamers for treating ocular diseases, hematologic disorders and cancer^226–228^.

In 2017, Matsui et al. screened DNA aptamers targeting RAGE in vitro, which could effectively inhibit the binding of AGEs to vRAGE, thereby markedly inhibiting renal NADPH oxidase activity and oxidative stress generation and attenuating the occurrence and progression of experimental diabetic nephropathy^229^. The research team subsequently applied AptRAGE in the treatment of various diseases. AptRAGE has demonstrated therapeutic efficacy in ameliorating deoxycorticosterone acetate/salt-induced renal injury in mice^230^, attenuating melanoma growth and hepatic metastasis in nude mice^231,232^, partially alleviating multiorgan damage in septic mice injected with LPS^233^, suppressing monocrotaline-induced pulmonary arterial hypertension progression in rats^234^, mitigating renal tubular injury and improving insulin resistance in diabetic mice^235^ and inhibiting colorectal cancer progression^236^.

RAGE aptamers have shown potential in inflammation, metabolic diseases and cancer, from early screening to multifunctional applications, but there are still many challenges in applying these aptamers in clinical applications. The stability of aptamers in vivo has always been a limiting factor in the effectiveness of drugs. The unmodified AptRAGE is easily degraded by nucleases. Although the screened AptRAGE has been phosphorothioated, its effect is further enhanced by other chemical modifications or liposome encapsulation to improve its stability. The targeting of aptamers needs to be further optimized to avoid cross-reactivity with RAGE isoforms (sRAGE) or other similar receptors (Toll-like receptors).

Future research on AptRAGE should focus on overcoming current bottlenecks in stability, penetration efficiency and scalable production through engineering optimization and interdisciplinary technologies (nanotechnology and artificial intelligence). The inherent advantage of high structural plasticity positions AptRAGE as a promising complement or alternative to antibodies in precision medicine. Strategic molecular engineering could enhance therapeutic efficacy by designing tandem or branched architectures to cotarget RAGE with disease-related molecules (VEGF and TNF-α), creating bifunctional/multivalent aptamers for synergistic effects, incorporating peptide chains or glycosylations to improve targeting specificity and tissue retention time and developing aptamer–nanomedicine hybrids through conjugation with liposomes, gold nanoparticles or exosomes to achieve spatiotemporally controlled delivery systems. Simultaneously, AptRAGE can demonstrate diagnostic potential through biosensor integration for the quantitative detection of sRAGE or RAGE ligands in biofluids (blood/cerebrospinal fluid), enabling early diagnosis of Alzheimer’s disease or sepsis; radiolabelled probes for PET imaging can localize inflammatory foci or neoplastic lesions.

### Other antagonists

Recent studies have revealed that multiple natural agents exert therapeutic effects on ovarian pathologies through RAGE signaling modulation. Erxian decoction can increase ovarian antioxidant/anti-apoptotic capacity by downregulating the expression of RAGE, PI3K, proapoptotic proteins (BAX and CASPASE3) and senescence markers (p16, p21, p53 and lamin A/C) while increasing anti-apoptotic BCL-2 expression. These coordinated actions restore estrous cyclicity and hormonal output, suggesting therapeutic potential for POF and granulosa cell senescence^237^. Acacetin, a plant polyphenolic compound, can attenuate LPA-mediated RAGE-PI3K-AKT signaling activation in the human peritoneal mesothelial cell coculture-induced malignant potential of ovarian cancer cells^176^. Naringin is a flavanoside that has been found to have a myriad of pharmacological benefits as well as antioxidant and anti-inflammatory properties. Naringin can ameliorate type 2 diabetes-associated steatohepatitis via the suppression of RAGE-NF-κB-mediated mitochondrial apoptosis pathways^238^. Geniposide specifically targets AGE–RAGE interactions through competitive V-domain binding. Zhu et al. characterized the hydrogen bond-mediated inhibition of AGE–RAGE complexation, effectively suppressing inflammatory cascades in diabetic nephropathy models—a mechanism potentially transferable to ovarian inflammatory pathologies^239^.

Other drugs that are commonly used to treat other diseases have also been found to be beneficial in the treatment of ovarian diseases. Adiponectin is an insulin-sensitizing hormone that can counteract D-galactose-induced granulosa cell senescence through multiple mechanisms. They reported its ability to reduce SASP-related proinflammatory cytokines (IL-6 and IL-8), preserve telomeric integrity and decrease SA-β-gal accumulation, collectively enhancing granulosa cell metabolic resilience^240^. Metformin restores ovarian homeostasis in D-gal-induced dysfunction models via PI3K–Akt–FOXO3a pathway activation. Ellibishy et al. reported marked reductions in follicular atresia and cleaved caspase-3 expression alongside normalized primordial follicle ratios and AGE levels, highlighting its pleiotropic therapeutic actions^28^. 1,25-Dihydroxyvitamin D3 may modulate AGE–RAGE signaling in human granulosa cells by downregulating RAGE expression, thereby attenuating AGE-induced suppression of steroidogenic gene expression^160^. Coenzyme Q10 (Mediterranean diet supplement) reduces postprandial oxidative stress and serum AGE levels in elderly populations by modulating RAGE expression and AGE metabolic pathways^241^.

The RAGE signaling pathway has emerged as a pivotal therapeutic target for ovarian pathologies, while the structural diversity and pleiotropic properties of natural agents unlock novel avenues for precision medicine with considerable translational potential. To advance clinical applications, future efforts should prioritize the following: (1) pharmacokinetic optimization: enhancing delivery efficiency of natural therapeutics through advanced systems to improve bioavailability. (2) Mechanistic elucidation: deciphering the spatiotemporal dynamics of RAGE signaling networks within the ovarian microenvironment to establish robust theoretical frameworks for targeted drug development and develop broad-spectrum therapeutics. (3) Long-term safety profiling: addressing critical gaps in current research by systematically assessing chronic exposure effects on ovarian reserve integrity and transgenerational developmental outcomes.

### Combination therapy with RAGE antagonists

Combination therapy against RAGE is also one of the means to further enhance its therapeutic effect. The seizure threshold in mice treated with a RAGE monoclonal antibody and TAK242, a TLR4 inhibitor, was markedly greater than that in the saline-treated group. Blocking the RAGE and TLR4 signaling pathways early after traumatic brain injury is a promising strategy for preventing post-traumatic epilepsy^242^. Unlike dexamethasone and S100A9 inhibitors, RAGE inhibitors do not reduce the efficacy of anti-PD-1 immunotherapy in glioma-bearing mice, providing an alternative treatment option for dexamethasone-induced postoperative cerebral edema^243^.

**Fig. 10:**
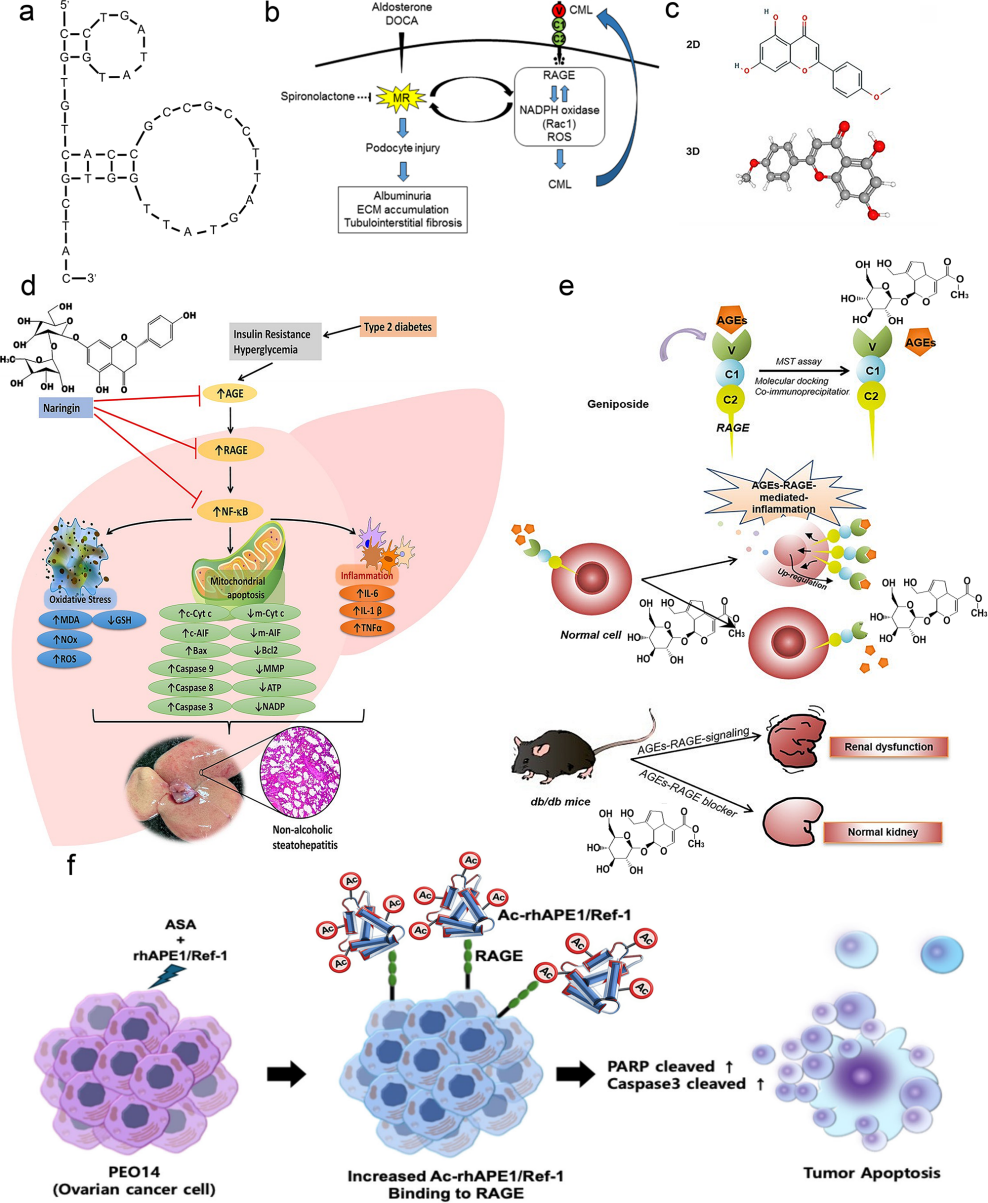
RAGE inhibitors. **a** Predicted secondary structure of clone #1 RAGE aptamer^229^. **b** Hypothetic pathways of Aldo–MR system and AGE–RAGE axis-mediated podocyte injury in hypertensive nephropathy^230^. **c** Chemical structure of acacetin^176^. **d** Naringin reduces T2DM-induced liver inflammation, oxidative stress and apoptosis^238^. **e** Geniposide effectively attenuates AGEs-induced RAGE activation by directly blocking AGE–RAGE signal transduction, thereby mitigating inflammatory responses^239^. **f** ASA induces rhAPE1/Ref-1 acetylation, increases RAGE expression and triggers apoptosis through APE1/Ref-1-RAGE interaction^177^.

The multitarget synergistic strategy is a popular research direction for current drug development, and future research should focus on the design of bifunctional antibodies or aptamers and combination chemotherapy drugs to further improve the therapeutic effect of RAGE drugs^176,177,229,230,238,239^ (Fig. 10).

## Discussion

The emerging recognition of RAGE as a pivotal molecular mediator in ovarian pathophysiology highlights its strategic positioning as a promising therapeutic target for both age-associated ovarian decline and pathological reproductive conditions. Current understanding of RAGE-mediated mechanisms in ovarian tissue remains understudied, with existing hypotheses primarily extrapolated from investigations in related physiological systems. This conceptual framework aims to stimulate innovative research directions for elucidating RAGE’s multifaceted roles in ovarian function regulation, particularly focusing on its implications for folliculogenesis, steroidogenesis and cellular senescence mechanisms.

In contemporary clinical practice for ovarian aging and related disorders, hormone replacement therapy maintains its status as the cornerstone intervention, complemented by adjuvant lifestyle modifications and nutritional supplementation^244,245^. Nevertheless, conventional therapeutic approaches are constrained by two principal limitations: (1) chronic adverse effects associated with prolonged pharmacotherapy, including thromboembolic events and potential carcinogenic risks^246^ and (2) substantial interpatient heterogeneity in therapeutic efficacy^247^. Emerging evidence highlights the pivotal role of RAGE, a master regulator of inflammatory cascades and oxidative stress responses, in accelerating follicular atresia and impairing ovarian steroidogenesis during aging processes. This pathological progression appears mediated through RAGE-dependent activation of multiple intracellular signaling networks. Current RAGE-targeted therapeutic strategies predominantly employ extracellular ligand blockade mechanisms, exemplified by sRAGE administration. However, this paradigm presents two critical limitations: (1) inherent incapacity to modulate RAGE-mediated intracellular feedforward amplification loops, such as NF-κB-induced transcriptional upregulation of RAGE expression^248^, and (2) unfavorable pharmacokinetic characteristics of sRAGE, particularly its abbreviated plasma half-life, which necessitates frequent dosing regimens and poses considerable challenges for clinical translation^249^. Further investigations indicate that RAGE-targeted therapies face the following scientific challenges: (1) complexity of RAGE signaling networks: RAGE interacts with multiple ligands (AGEs, HMGB1, S100 and so on), and its downstream pathways involve cross-regulatory mechanisms encompassing inflammation, autophagy and apoptosis, limiting the efficacy of single-target approaches. (2) Tissue-specific challenges: systemic RAGE inhibition may interfere with physiological repair processes (wound healing), necessitating the development of ovary-specific targeted delivery technologies. (3) Lack of dynamic monitoring tools: the dynamic correlation between ovarian aging biomarkers and RAGE activity remains unclear, hindering therapeutic efficacy evaluation.

To overcome these bottlenecks in RAGE-based drug development, future research directions should prioritize: (1) Multitarget synergistic interventions: given the maturity of RAGE small-molecule inhibitors, combining them with antioxidants or mitochondrial protectants (coenzyme Q10) to simultaneously target the inflammation-oxidative stress axis. Emerging research highlights the therapeutic potential of combinatorial strategies to address the complex pathogenesis of ovarian diseases, including POI, PCOS and malignant ovarian tumors. (2) Epigenetic regulation integration: utilizing histone deacetylase inhibitor to correct ovarian aging-associated DNA methylation abnormalities. (3). Precision delivery systems: developing exosome- or nanoparticle-encapsulated drugs conjugated with ovarian homing peptides (FSHR-targeting peptides) to minimize systemic exposure, designing stimuli-responsive release systems (ROS-sensitive nanocarriers) for localized drug delivery in ovarian microenvironments with high oxidative stress. (4) Biomarker-guided personalized therapy: establishing multiomics predictive models integrating ovarian aging biomarkers (genomic instability, chronic inflammation and so on) to identify RAGE-high subpopulations for stratified treatment. Creating real-time RAGE activity monitoring technologies (PET tracers) for dynamic therapeutic adjustment.

Over the next 5–10 years, ovarian aging treatment is expected to shift toward multidimensional modulation of ‘inflammation–metabolism epigenetics’. Functioning as a pivotal nexus that orchestrates oxidative stress signaling, RAGE-targeted interventions demand collaborative integration of molecular biology, bioengineering, and clinical oncology expertise to realize their complete therapeutic potential.

## Data Availability

This Review Article does not involve the creation or analysis of original data. All referenced data are available in the respective published sources, which are cited throughout the manuscript.
